# The phi027 bacteriophage influences physiology and virulence of the lysogenic strain of *Clostridioides difficile*

**DOI:** 10.1038/s41598-025-04106-0

**Published:** 2025-05-29

**Authors:** Natalia Frankowska, Klaudia Szarek, Adam Iwanicki, Dorota Wultańska, Hanna Pituch, Monika Kabała, Alessandro Negri, Michał Obuchowski, Krzysztof Hinc

**Affiliations:** 1 Division of Molecular Bacteriology, Medical University of Gdańsk, Gdańsk, Poland; 2 Intercollegiate Faculty of Biotechnology, University of Gdańsk, Gdańsk, Poland; 3 Department of Medical Microbiology, Faculty of Medical Science in Katowice, Medical University of Silesia, Katowice, Poland; 4 Department of Medical Microbiology, Medical University of Warsaw, Warsaw, Poland

**Keywords:** Bacteriophage, *Clostridioides difficile*, CRISPR-Cpf1, Sporulation, Adhesion, Cytotoxicity, Bacterial host response, Bacteriophages

## Abstract

*Clostridioides difficile*, the causative agent of *C. difficile* infections (CDI), can be naturally infected by bacterial viruses known as bacteriophages. All characterized bacteriophages of this bacterium are temperate, meaning that upon infection their genetic material integrates and replicates with host’s genome. Such lysogenic strains can exhibit altered physiology and virulence, which in turn can be an important factor for epidemiology of CDI. In this study we characterized the phiCDKH02 bacteriophage infecting clinical isolates of *C. difficile* belonging to hypervirulent ribotypes 027 and 176. The bacteriophage was found to be identical to phi027. To get some insight into the role of this bacteriophage in physiology of its host and interaction with human colon cells, we made use of CRISPR-Cpf1 technology to cure the lysogenic *C. difficile* of the prophage. The prophage-free strain exhibited altered sporulation efficiency, lowered adhesion and decreased cytopathic effects towards human colon cells associated with decreased production of TcdB. These results emphasize importance of prophages in shaping virulence of *C. difficile*.

**Supplementary Information:**

The online version contains supplementary material available at 10.1038/s41598-025-04106-0.

## Introduction

*Clostridioides difficile* is a Gram-positive, anaerobic bacterium and one of the most important human pathogens responsible for nosocomial acquired diarrhea^1^. *C. difficile* infections (CDI) follow dysbiosis of microbiota caused by antibiotic treatment^2^. The most severe cases of CDI lead to development of pseudomembranous colitis and fulminant colitis^3^. The capability of *C. difficile* to form spores contributes to increased recurrent infections, as well as makes elimination of these bacteria from hospital environment especially difficult.

Major virulence factors of *C. difficile* are toxins: toxin A (TcdA), toxin B (TcdB) encoded in the PaLoc pathogenicity locus. Some strains produce binary toxin (CDT) encoded by *cdtA* and *cdtB* genes located on chromosome^4,5^. Production of these three toxins, is the characteristic feature of the hypervirulent strains which were shown to cause more severe CDI. Such strains additionally can exhibit high sporulation capacity and resistance to fluoroquinolones^6,7^. *C. difficile* strains are classified to certain PCR-ribotypes (RT-ribotypes) based on pattern of PCR products obtained in reactions amplifying the intergenic spacer (ITS1) regions between rRNA 16 and rRNA 23 genes encoding rRNA^8^. The most commonly known hypervirulent RT027, also identified as the BI/NAP1/027, contributed to spread of this pathogen in both, Europe and North America^9^. Apart from RT027 the RT078 was also described as the hypervirulent^10^.

*C. difficile*, similar to bacteria of other species, can be infected with viruses, commonly known as bacteriophages (reviewed in^11^. All *C. difficile* bacteriophages identified so far are temperate which means that upon infection of host cell their genetic material integrates and replicates with host’s genome. Induction of the prophage can be caused spontaneously^12^ or following cellular stressors, mainly those leading to the DNA damage^13^. The presence of prophages in the genome can influence the physiology and virulence of the host. It is worth noting that this influence may be both, negative and positive. The example of the first is phiCD119 phage which has been shown to reduce toxin production^14,15^. This effect can be accounted for production of a specific phage repressor RepR which binds upstream of genes encoding toxins in the PaLoc and decreases their transcription^14^. The decrease of toxin production has also been observed for *C. difficile* strains lysogenic with phiCD27 phage^16^. The opposite effect is caused by phiCD38-2 phage on its host. In the case of bacteria harboring this bacteriophage, the increased production of both toxin A and toxin B was observed^17^. Other phages, such as phiC2, phiC5 and phiC6, cause increased production of toxin B by the lysogenic hosts^18^. Apart from the influence on toxin production prophages can change the expression of *C. difficile* surface proteins^19^, supposedly interfere with quorum sensing^20^, and change the host’s susceptibility to antibiotics^21^.

In this study we induced and isolated a phage from Polish clinical *C. difficile* strain 500/12. The phage is identical to the previously characterized phi027 bacteriophage^22–24^. Interestingly, we found this phage in genomes of 14 other *C. difficile* strains obtained from clinical samples, as well as its presence was confirmed in genomic sequences of several dozen *C. difficile* strains available in the GenBank database. The curing of the lysogenic strain R20291 of this prophage was reported previously by Hussain et al., although the removal was not perfect^24^. Therefore we applied different CRISPR system to cure the 500/12 strain of the prophage and compared the characteristics of both, lysogenic and prophage-free strains. The prophage-free strain exhibited significantly lower virulence towards human colon cells suggesting an important influence of phi027 on the properties of *C. difficile* lysogenic with this phage.

## Results

### Detection and isolation of phage from *C. difficile* strain 500/12

The *C. difficile* strain 500/12 originates from the collection of strains of the Department of Medical Microbiology of the Medical University of Warsaw. It is one of the first strains identified in Poland with the PCR ribotype 176^22^. According to the former classification of *C. difficile* phages two families of viruses were distinguished: *Siphoviridae* and *Myoviridae* (both now reclassified as *Caudoviricetes*). The 500/12 strain was therefore PCR screened for presence of possible prophages in its genome using primers targeting the holin genes of both mentioned phage families. Upon detection, the prophage was induced from the host’s genome using mitomycin C (Fig S1). The myovirus holin gene was amplified from obtained phage lysate confirming the presence of a virus belonging to this family (data not shown). The phage was initially named phiCDKH02. (accession no. PP767789).

### The phage phiCDKH02 induced from 500/12 strain is identical to phi027 bacteriophage

**Fig. 1:**
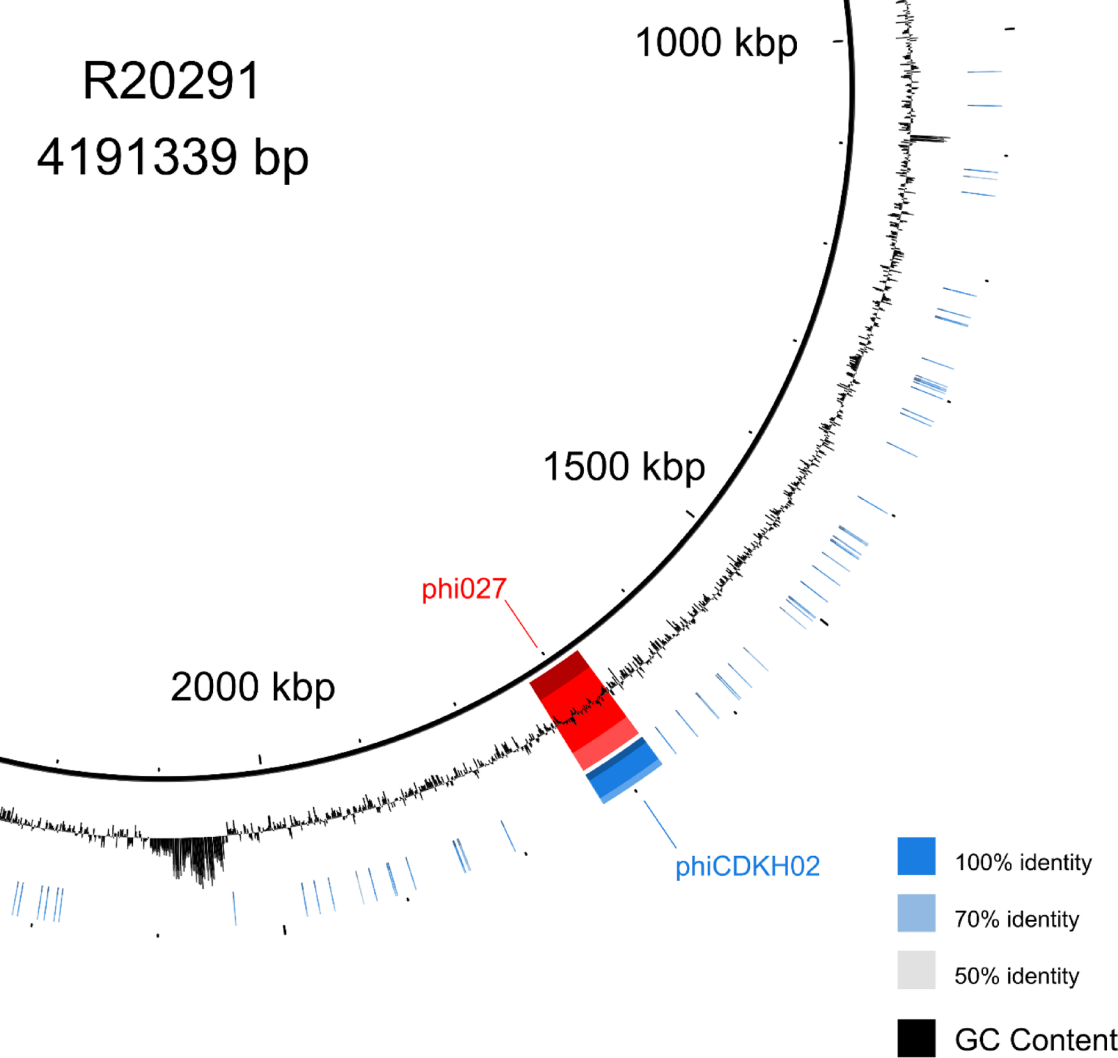
BLAST Ring Image Generator (BRIG) image of comparison of phiCDKH02 and phi027 prophage located in *Clostridioides difficile* R20291.

Genomic DNA of the induced phiCDKH02 was purified and sequenced. At the same, time the whole genome sequencing of its host strain 500/12 was performed. Obtained genomic sequences were annotated and deposited in the GenBank (phiCDKH02 accession no. PP767789, 500/12 accession no. CP180209). BLAST search of sequence database performed with phiCDKH02 genomic sequence indicated that it is identical to the sequence of the phi027 prophage present in the strain R20291^24^ (Fig. 1). Features of the phage genome were visualized in the Fig S2 and listed in the Table S1, along with predicted protein products.

The identified prophage was the only intact one in the genome 500/12 strain, as suggested by the sequence analysis performed with PHASTER^25,26^. Comparison of genomic sequences of strains 500/12 (RT176) and R20291 (RT027) suggests very high level of their identity in spite of the assignment to different ribotypes. Nonetheless, 500/12 genome lacks a predicted mobile element present in position 2,043,583–2,087,750 of the R20291 genome.

### Identification of phi027 prophage in clinical isolates

**Fig. 2:**
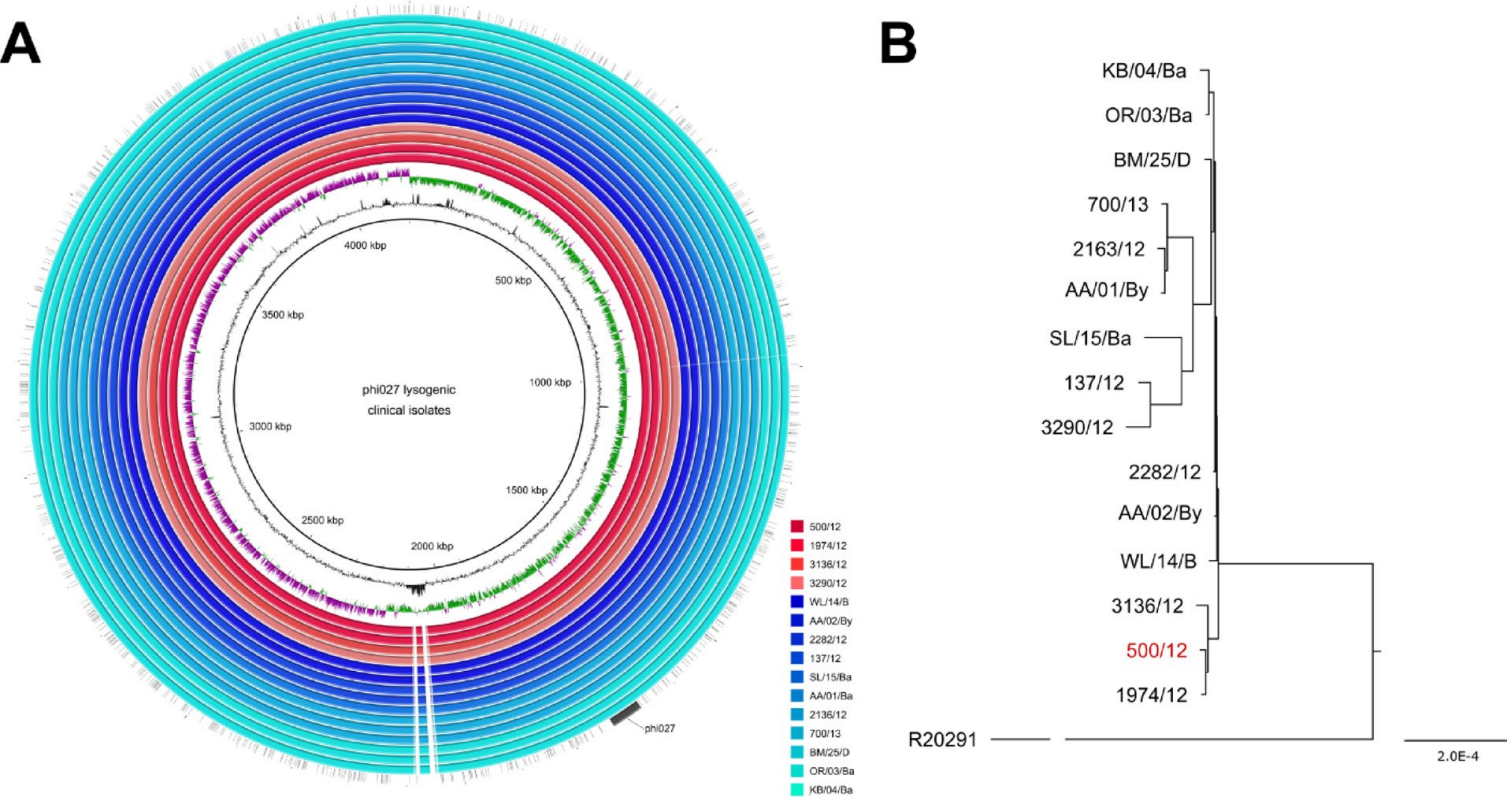
Comparative phylogenetic analysis based on genomic sequences of clinical strains harboring phi027 prophage isolated from three Polish hospitals. **(A)** BLAST Ring Image Generator (BRIG) image of comparison of genomic sequences of clinical isolates lysogenic with phi027. R20291 genome was used as a reference. Red colors – strains ribotyped as RT176, blue colors – strain ribotyped as RT027. The innermost circles represent the GC content (black) and GC skew (purple/green) of the R20291 chromosome. **(B)** The guide tree generated using the progressive Mauve algorithm. Strain 500/12 used for isolation and characterization of the prophage identical to phi027 indicated in red. R20291 branch has been shortened for better clarity of the figure.

Based on the genomic sequence of phage isolated from 500/12 strain we designed two pairs of primers enabling specific detection of this phi027 prophage in genomes of *C. difficile*. Upon PCR screening of a collection of clinical isolates belonging to RT027 and RT176 we were able to identify another 14 strains harboring this prophage (Table S2). The genomes of these isolates were sequenced and in subsequent analysis, alike to 500/12, we identified the full sequence of phi027. Moreover, alignment of genomic sequences performed with progressive Mauve algorithm led us to the conclusion, that all of these isolates exhibit very high identity of genomes (Fig. 2). The estimated ratio of changes in nucleotide sequences between genomes of a particular strain and 500/12 ranged from 0.07 to 1.29 per 1000 bp. The protein sequences encoded in these genomes exhibited between 0.03 and 0.83 ratio of changes per 1000 amino acid residues.

### The phi027 prophage is present in other hypervirulent strains of *C. difficile*

**Fig. 3:**
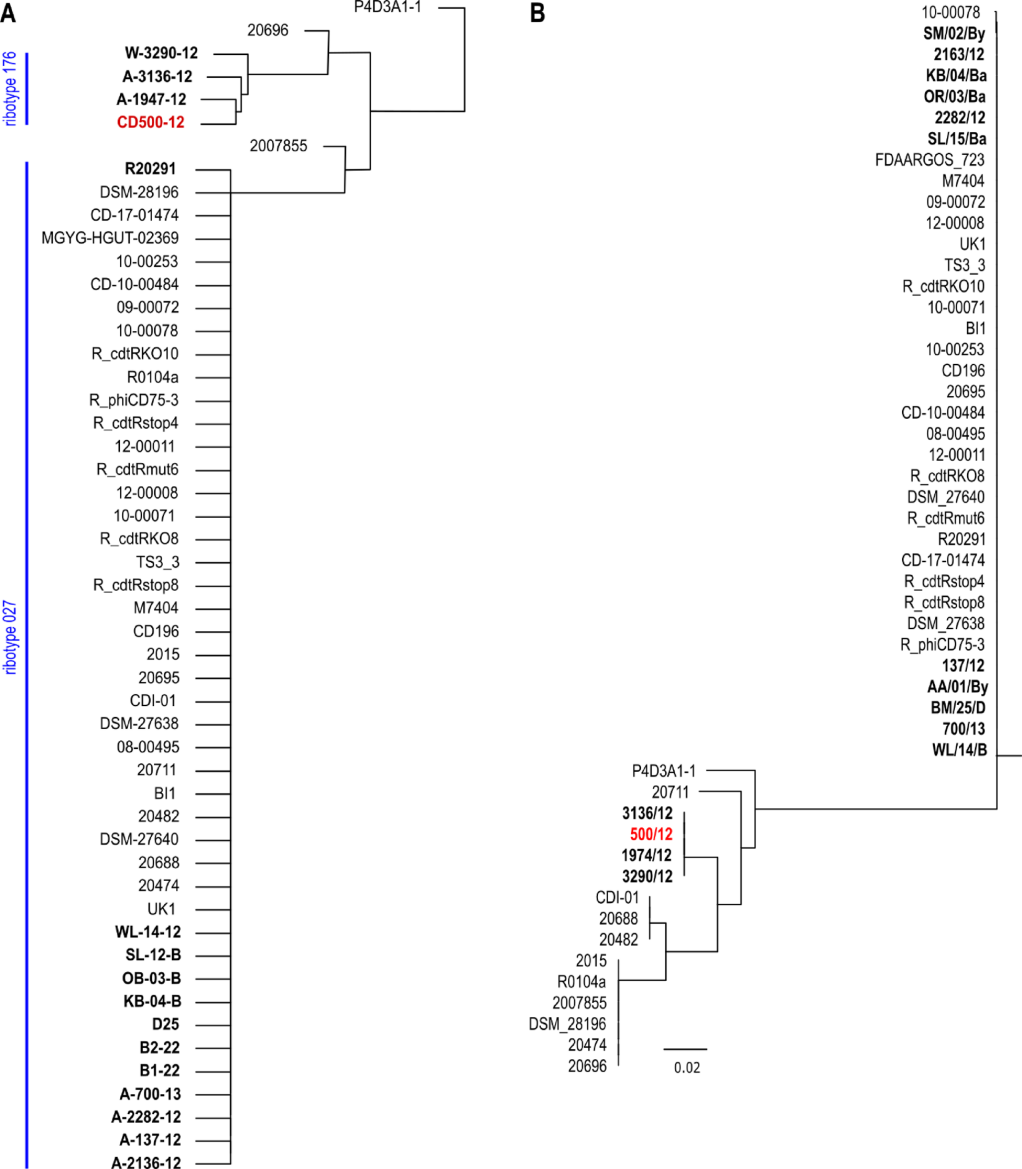
**(A)** Hierarchical clustering of in silico ribotyping results. Strains with confirmed ribotypes indicated in bold. Strain *C. difficile* 500/12 used for isolation and characterization of the prophage identical to phi027 indicated in red. **(B)** Maximum likelihood tree of phi027 lysogenic *Clostridioides difficile* strains. Strain 500/12 used for isolation and characterization of the prophage identical to phi027 indicated in red. Clinical strains isolated in this study in bold.

We used the full sequence of phi027 bacteriophage to perform a BLAST search of sequence databases. We found 37 genomes of strains harboring the complete sequence of the prophage exhibiting 100% of identity. Detailed analysis of these genomes led us to the conclusion that they are highly identical, even though strains were collected at different times and areas of the world (Table S4). Moreover, three of them were genomic sequences of the R20291 strain (GenBank accession numbers CP029423, CP115183, FN545816). Since we had no information regarding ribotypes of these strains we performed in silico PCR with primers used for ribotyping of *C. difficile*. Obtained patterns of hypothetical PCR products were compared with the results of such analysis done for genomic sequences of clinical isolates used in this study. Using such approach, we were able to assume that the majority of analyzed strains most probably belong to hypervirulent ribotypes 027 and 176 (Fig. 3A). The ratio of changes in nucleotide sequences between analyzed genomes and 500/12 genome varied from 0.68 to 1.44 per 1000 bp. In the case of protein sequences, the amino acid residues change ratio was between 0.24 and 3.17 per 1000 residues. To investigate in depth the phylogenetic relationship between these strains we used amino acid sequences of seven selected open reading frames (Table 1), which exhibited the highest variability between analyzed strains and 500/12 to generate a maximum likelihood tree (Fig. 3B).

**Table 1:** Proteins selected for Building a maximum likelihood tree of phi027 lysogens.

Protein_id	Product
AJP10080.1	putative cell surface protein
AJP13166.1	putative cell surface protein
AJP10067.1	putative membrane protein
AJP10068.1	putative ATPase
AJP13155.1	putative ATPase
AJP13154.1	putative conjugative transposon protein

### The phi027 bacteriophage influences the host’s properties

**Fig. 4:**
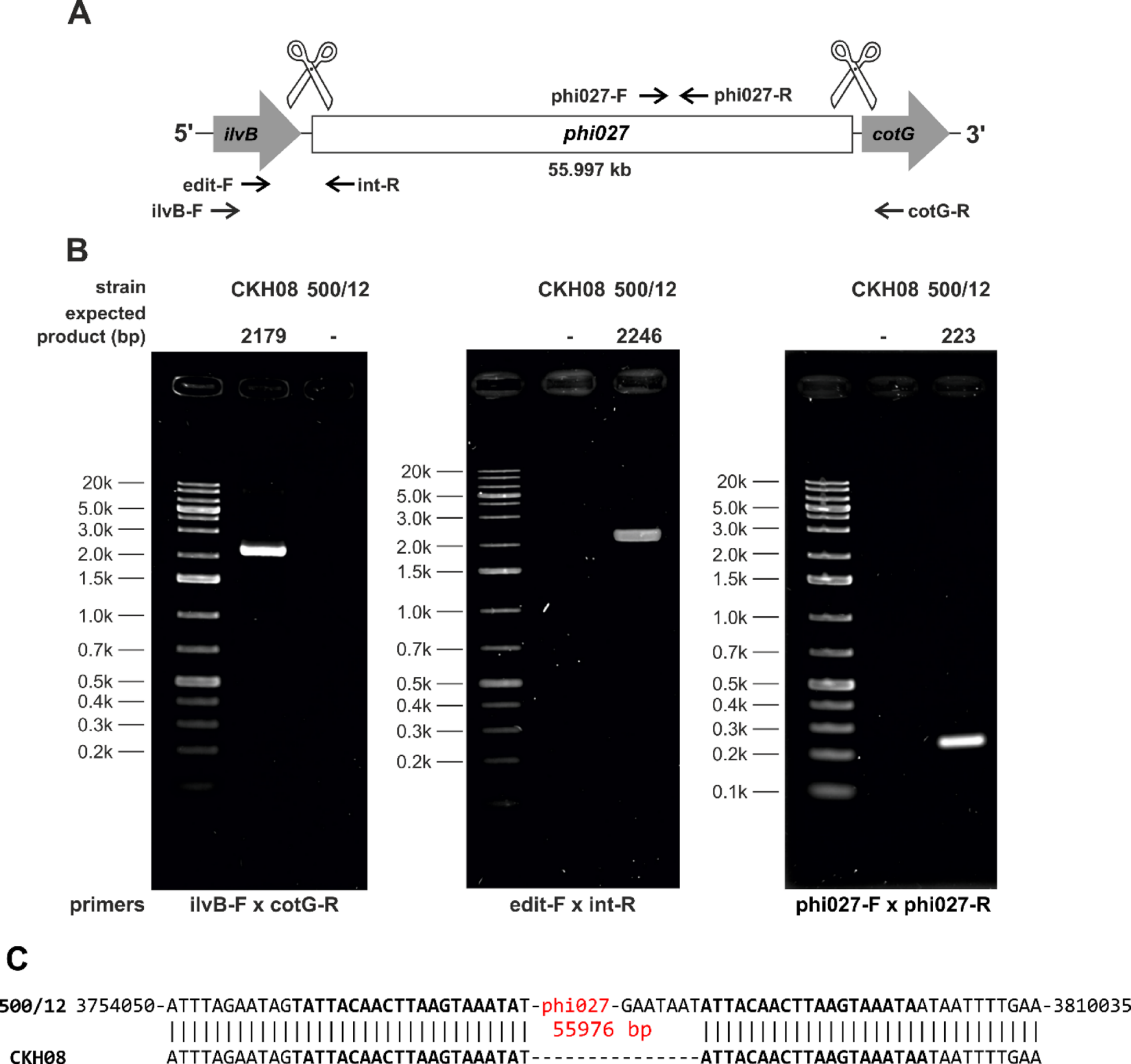
CRISPR-Cpf1 deletion of phi027 prophage from strain 500/12. **(A)** Schematic drawing of prophage deletion. Arrows indicate positions of primers used in PCR reactions whose products were separated by electrophoresis. **(B)** PCR products obtained in reactions run with genomic DNA of strains CKH08 and 500/12 as template and colony PCR of strain CKH08 and 500/12 (rightmost panel) separated by agarose gel electrophoresis. Expected sizes of PCR products indicated above the gels. Primers used in PCR reactions denoted below the gels. **(C)** Sanger sequencing of phi027 prophage deletion region in the genome of CKH08 strain. Coordinates marked at the genomic sequence of the strain 500/12 provided according to the sequence JBCJLD000000000. DNA sequences of the *attL* and *attR* sites^19^ in bold.

**Fig. 5:**
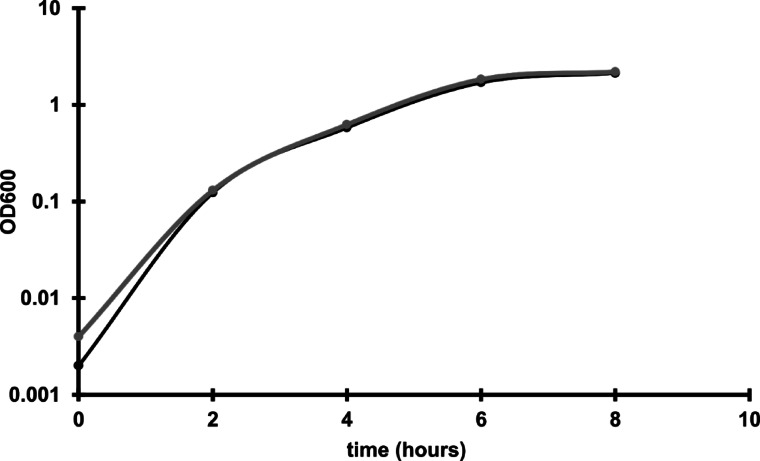
Growth of 500/12 (black line) and CKH08 (grey line) in BHI medium.

**Fig. 6:**
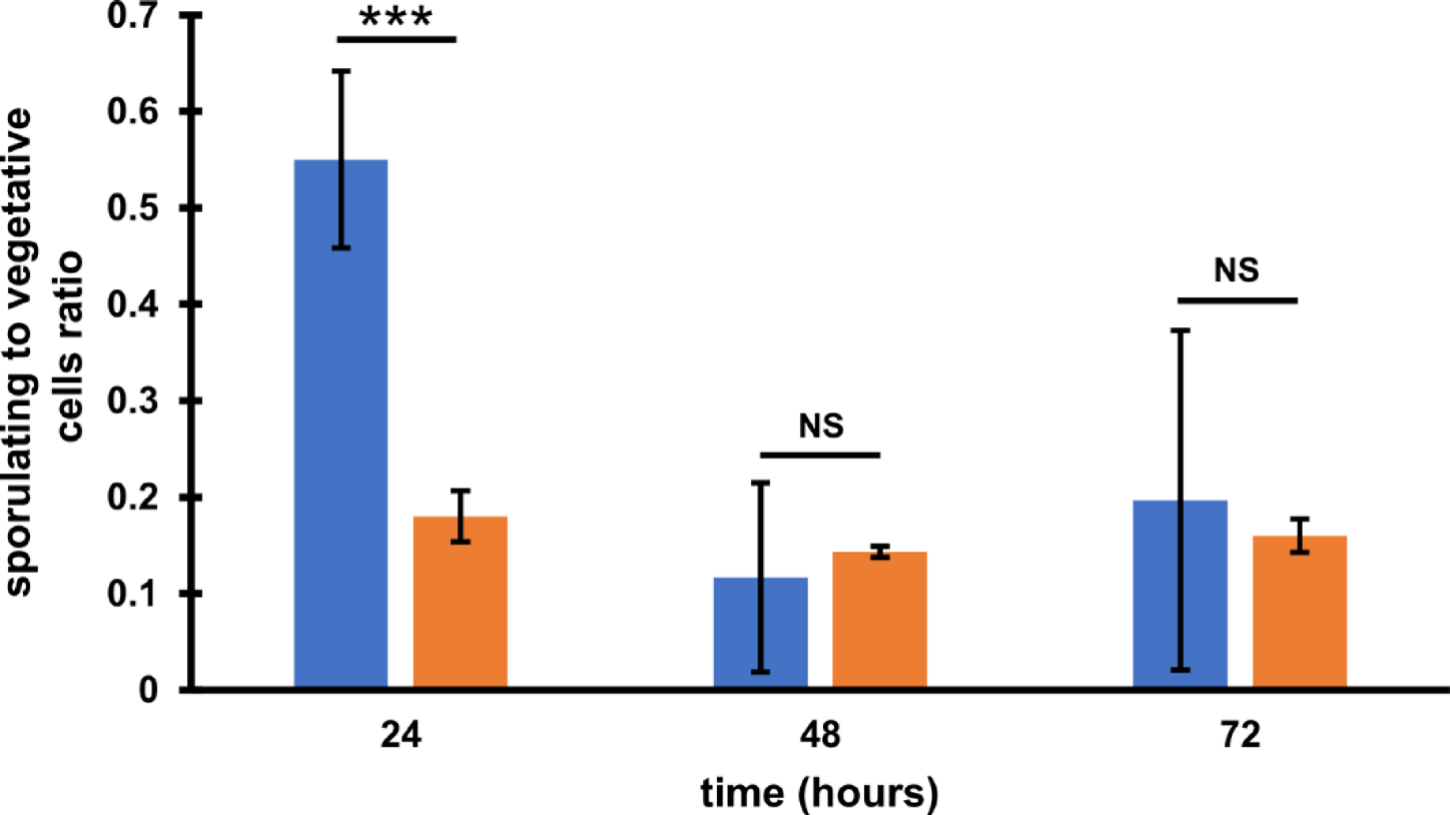
Sporulation efficiency expressed as the spores to vegetative cells ratio within a population. Blue bars – 500/12, orange bars – CKH08. Ratios represent means of three repeats (*n* = 3) of the experiment. Error bars indicate standard deviations. *** *p* < 0.001, NS – not significant.

Since the phi027 prophage was detected in genomes of hypervirulent strains we wanted to verify whether it can influence its host and contribute to its virulence. To verify this, we cured 500/12 strain for the prophage using the CRISPR method^27^(Fig. 4). The obtained strain was named CKH08. Analysis of the genomic sequence of the cured strain obtained after whole genome sequencing (CHK08 accession no. CP180211) confirmed that the CRISPR-Cpf1 editing resulted in removal of the phi027 prophage and did not cause other changes. Additional Sanger sequencing of the phi027 prophage integration region of the cured strain confirmed its complete deletion (Fig. 4C). To ensure that the phi027 phage did not exist in CHK08 as a phagemid we performed colony PCR reactions with phage-unique primers phi027-F and phi027-R targeting ORF *phiCDKH02_43* of the phage. No PCR products obtained for the cured strain confirmed lack of phi027 phage in the analysed strain (Fig. 4AB). The cured strain exhibited growth efficiency (Fig. 5) and biofilm formation (Fig S3) similar to the parental 500/12 (data not shown). Interestingly, the spore formation kinetics of the CKH08 strain was different than that observed for the 500/12 (Fig. 6). The ratio of sporulating to vegetative cells upon 24 h of incubation surpassed 0.5 in the case of 500/12 strain suggesting that more than half of cells initiated the sporulation process. At the 48 and 72-hour timepoints this ratio dropped to 0.1 and 0.2, respectively and exhibited much higher variability. In the case of CKH08 strain the ratio of sporulating to vegetative cells only slightly exceeded 0.2 and remained at the same level after 48 and 72 h of incubation. Taken together these results suggest that the presence of phi027 prophage in the host genome positively influences its capability of initiating sporulation.

**Fig. 7:**
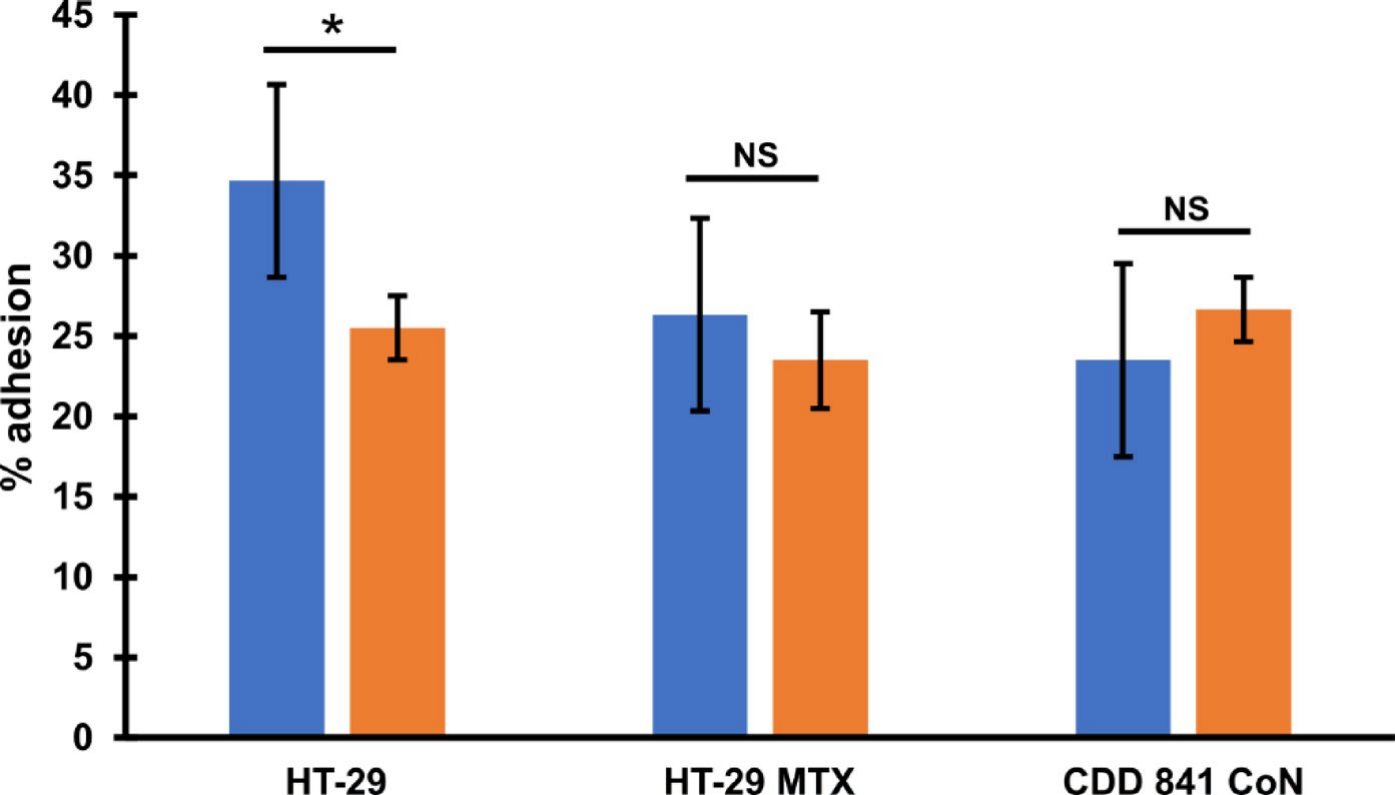
Adhesion of *C. difficile* to human colonic cells. *C. difficile* strains 500/12 (blue bars) and CKH08 (orange bars) were used to analyze adhesion to cells of lines HT-29, HT-29 MTX (mucous-secreting) and CDD 841 CoN. The percentage of adhesion represent the means of six repeats (*n* = 6) of the experiment. Error bars indicate standard deviations. * *p* < 0.01, NS – not significant.

Pathogenesis of *C. difficile* infection relies on efficient colonization of the host’s colon^28^. Because of that, we wanted to check how the presence of phi027 prophage influences adherence of *C. difficile* to colonic cells. We analyzed how cells of 500/12 and CKH08 adhered to HT-29 human colorectal adenocarcinoma cells and their mucous-secreting subclone HT-29-MTX, as well as to CCD 841 CoN human normal colon epithelial cells. Cells of CKH08 strain exhibited significantly decreased adherence to non-mucosa HT-29 cells in comparison to bacteria of 500/12 strain (Fig. 7). Adherence to other cell lines was comparable for both analyzed *C. difficile* strains.

**Fig. 8:**
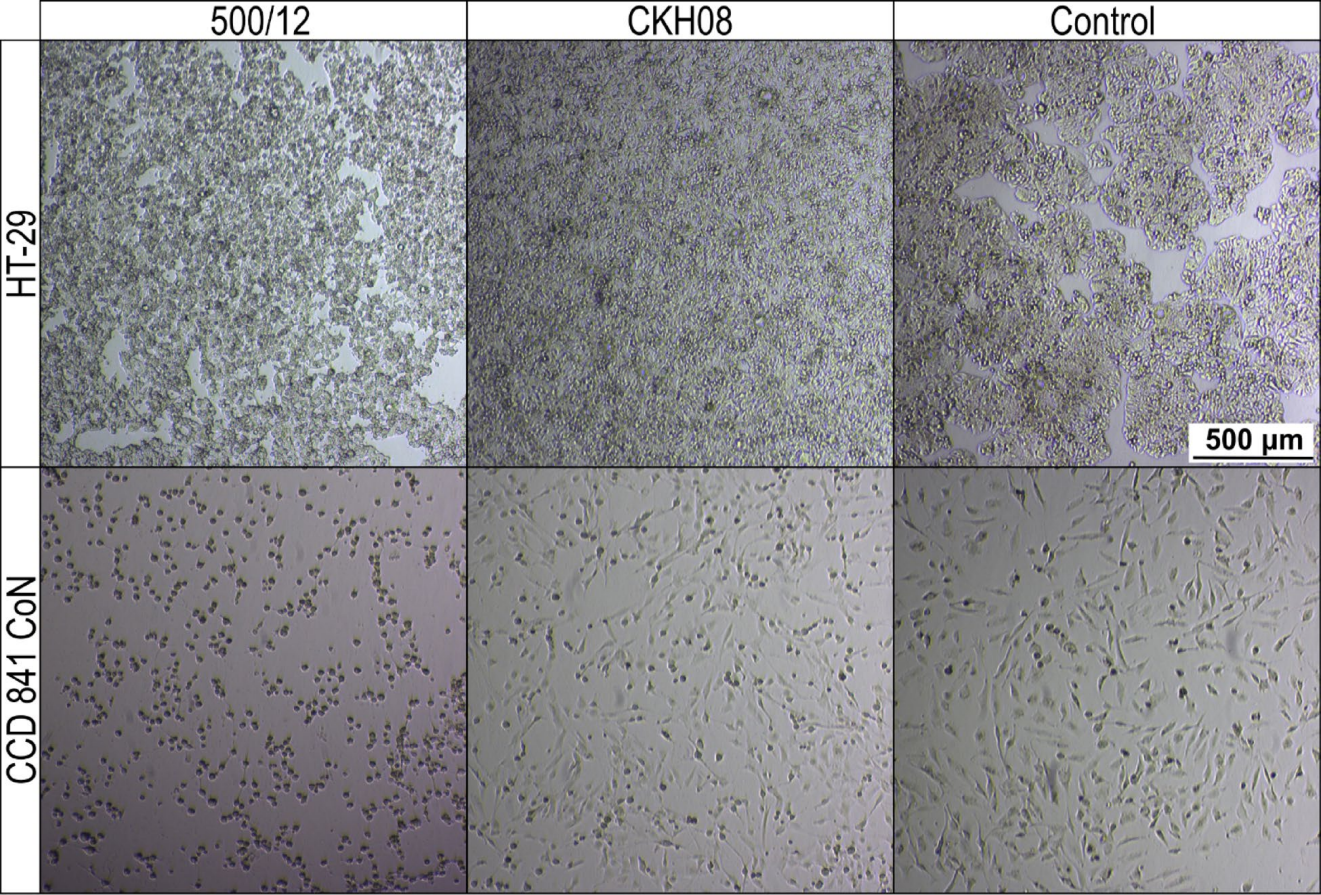
Cytopathic effect (CPE) of *C. difficile* culture supernatants on human colonic cells. CCD 841 CoN and HT-29 cells were treated for 48 h with 10^− 6^ dilution of culture supernatants of strains 500/12 and CKH08. Control cells were incubated with the addition of 500/12 supernatant neutralized with antitoxin B.

In the next step, we verified the cytotoxic effect of *C. difficile* towards investigated cell lines. For both, HT-29 and CCD 841 CoN cells we observed evidently lower cytotoxicity of CKH08 culture supernatant as compared to supernatant of 500/12 culture. Upon 24-hour incubation, 10^− 4^ dilution of CKH08 supernatant was required to elicit the same cytotoxic effect as the 10^− 6^ dilution of 500/12 supernatant. This difference decreased by one order of magnitude when the incubation was prolonged for another 24 h (Fig. 8; Table 2). The supernatants of strains 500/12 and CKH08 were diluted from 10^− 1^ to 10^− 8^ and all these dilutions were applied simultaneously to the cell lines. All of them were incubated for 24 h and 48 h. The titer of the cytopathic effect was considered to be the dilution in which there were 50% or more changed cells for a given strain and the reading was taken after 24 and 48 h (Fig. 8; Table 2). This led us to conclude, that the presence of phi027 prophage contributes to the increased cytotoxicity of its host.

**Table 2:** Comparison of cytotoxic effects of phi027 lysogenic and prophage-free strains of *Clostridioides difficile* on human colonic cells.

Strain	HT29		CCD 841 CoN	
	24 h	48 h	24 h	48 h
500/12	10^− 6^	10^− 6^	10^− 6^	10^− 6^
CKH08	10^− 4^	10^− 5^	10^− 4^	10^− 5^

Numbers come from representative experiments of three repeats (*n* = 3) and indicate dilutions of *C. difficile* culture supernatants used to treat colonic cells which resulted in the observation 50% of cytopathic cells.

**Fig. 9:**
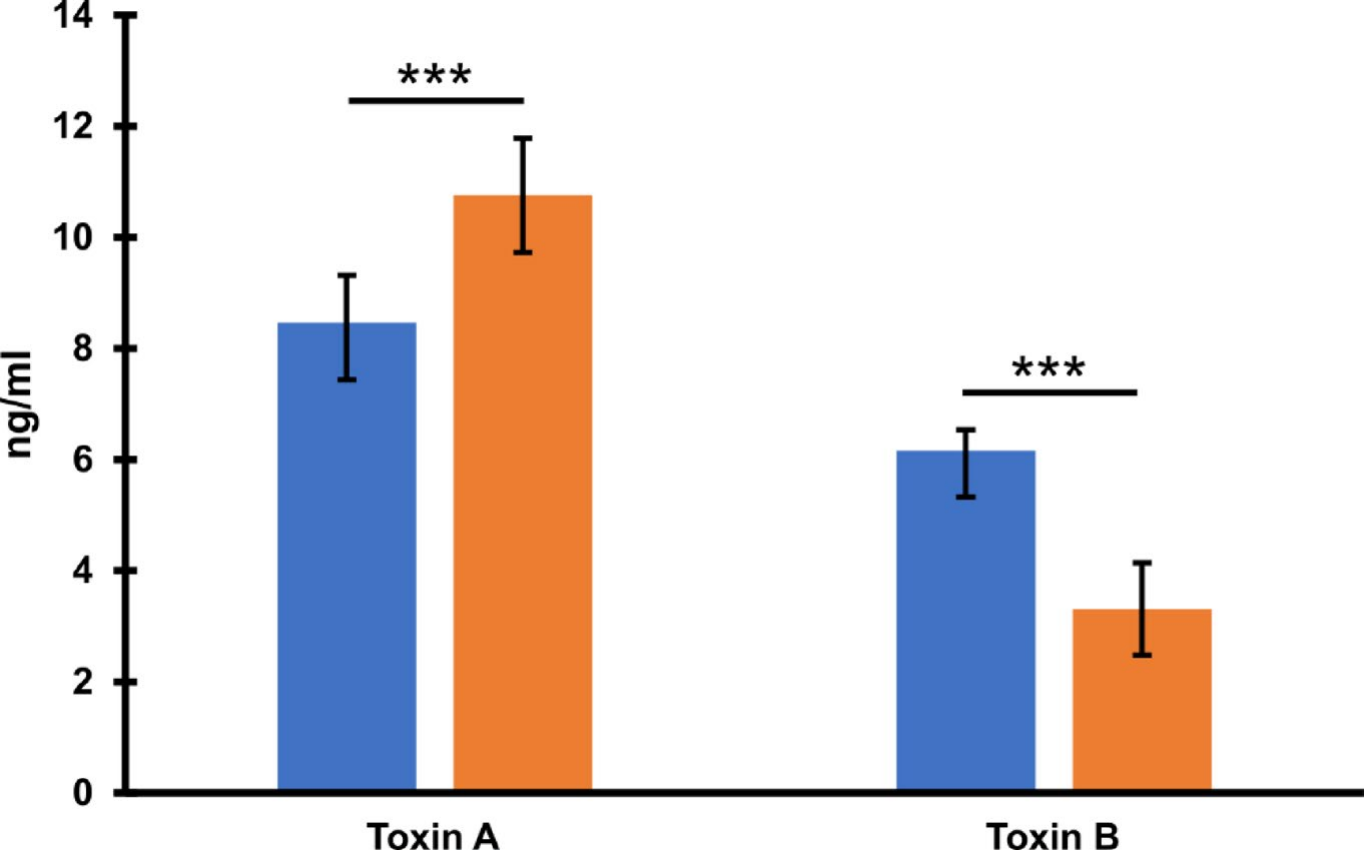
Levels of toxins A and B produced by *C. difficile* after 48-hour incubation. Blue bars – phi027 lysogenic strain 500/12, orange bars – prophage-free strain CKH08. Data represent means of 8 repeats (*n* = 8). Error bars indicate standard deviations. *** *p* < 0.001.

To justify the role of clostridial toxins in observed cytopathic effect we titrated toxins A and B in 48-hour culture supernatants of investigated strains (Fig. 9). The level of toxin A produced by the prophage-free CKH08 was increased in comparison to the phi027 lysogenic strain 500/12. On the other hand, the level of toxin B produced by CKH08 dropped nearly by half as compared to the 500/12. These results confirm importance of toxin B for decreasing the cytopathic effect observed in the case of colonic cells treated with supernatants of cells of the prophage-free strain (Fig. 8; Table 2) and collineate with inhibition of cytotoxicity in control cells treated with antitoxin B (Fig. 8).

## Discussion

Bacteriophages can influence the physiology of their hosts^29^. This phenomenon is especially important in the case of pathogens in which the presence of a prophage in the genome can contribute to increased virulence^30^. *C. difficile* is not an exception, since several phages infecting these bacteria were shown to lead to increased production of toxins^17,18^, which in turn influenced pathogenic properties of these bacteria.

Implementation of the consortium project of three Polish medical universities (Medical University of Gdańsk, Medical University of Warsaw and Medical University of Silesia) resulted in the collection and characterization of more than 400 clinical isolates of *C. difficile*. The functional prophage present the isolate 500/12^22^ which was originally named phiCDKH02, turned out to be identical to the already characterized bacteriophage phi027^23,24^. Interestingly, the original strain R20291 harboring this prophage was classified as the RT027 ^24^, while the strain 500/12 was characterized as the RT176, even though both strains share very high level of sequence identity. Another 14 isolates from our collection were also shown to harbor phi027 prophage in their genomes at the previously characterized *attB* site^19^. It is important to note, that these strains were isolated at different times and places in Poland. Moreover, while the phage sequence is entirely identical, the genomic sequences of these strains exhibit some differences. They are also divided into two ribotypes, 027 (8 strains) and 176 (4 strains). Very similar observation results from the search of the GenBank database. 37 identified genomic sequences of *C. difficile* contain entirely identical sequence of the entire phi027 prophage, including the R20291 strain. All 37 strains share high level of identity of genomic sequences, even though the time and geographic location of their isolations are different (Table S4). The 176 ribotype was suggested to be related to 027 ribotype based on the fact that it belonged to the same multilocus sequence type (ST1/clade 2) and the similarity of proteome profiles^31,32^. Although analyzed strains were presumably classified to the closely related ribotypes (Fig. 3A) their genomic sequences are not identical, which is in contrast to the identity of phi027 prophage sequence and location in their genomes. The explanation of such phenomenon can be that the bacteriophage propagates in the population of R20291-related stains rather than the phi027 lysogenic strain spreads in the environment. If the latter possibility was true the accumulation of changes should be observed in the genome of the phi027 prophage present in different *C. difficile* strains.

Our study on the influence of phi027 bacteriophage on the physiology of its host was possible due to obtaining a prophage-free version of the strain 500/12. The CRISPR-Cpf1 technology^27^ proved to be especially useful in performing nearly clean deletion of phi027 prophage from the genome of 500/12. Interestingly, Hussain et al.. performed similar manipulation of the phi027 host genome applying the CRISPR-Cas9 system, nevertheless, the curing of the strain of this prophage was not perfect, since the process of genome editing resulted in the deletion of the bacterial gene located downstream of the *attR* site and introduction of 2.7 kb remnant of the deletion plasmid^24^. Whole genome sequencing of the strain CKH08 and Sanger sequencing of the prophage integration confirmed deletion of the entire phi027 apart from 20 bp of the *attL* sequence (Fig. 4).

The increased probability of infection with phi027 bacteriophage seems to be beneficial to its host. Presence of the prophage in the genome does not change growth or biofilm formation capabilities of the lysogenic strain. On the other hand, the strain CKH08 cured of the phi027 prophage showed the lowered number of cells initiating the sporulation process (Fig. 5). The *C. difficile* gene *cotG* located downstream the *attR* sequence of the analyzed prophage encodes protein similar to *Bacillus subtilis* CotJC which localizes to the inner coat of the spore^33^. Since integration of the phi027 prophage might alter expression of this gene it is plausible to hypothesize that removal of the prophage could influence spore properties. Nonetheless, mutant strains of *B. subtilis* which do not incorporate this protein into the spore coat exhibit no apparent defects^33^, therefore, similar effects could be expected for *C. diffcile*. The ability to efficiently form spores contributes to the ease of *C. difficile* transmission. This fact is emphasized by the extraordinary resistance of *C. difficile* spores to most disinfectants, antibiotics, and, what is very important in the case of anaerobes, the effects of oxygen^34,35^. Above mentioned properties of phi027 lysogenic strains can increase their fitness and in turn contribute to enhanced spread in the vulnerable populations.

Another two very important features of phi027 lysogenic strain 500/12 are its increased adhesion (Fig. 7) and cytotoxicity (Fig. 8; Table 2) exhibited towards the selected intestinal cells. One of the crucial steps in *C. difficile* infection is colonization of the colonic niche^36^. Many surface proteins produced by this bacterium may contribute to this process^37^. Moreover, clostridial binary toxin CDT was shown to be important in promoting cytoskeleton rearrangement which, in turn, may enhance adhesion of bacteria to host epithelial cells^38^. The presence of phi027 prophage in the genome may influence the expression of genes encoding these proteins resulting in enhanced interaction of *C. difficile* with host tissue. In light of this, the decreased adhesion of phi027-free strain CKH08 may suggest better chances of successful colonization of the host by the bacteriophage-harboring bacteria. Moreover, increased toxicity of a strain lysogenic with phi027 prophage may also contribute to more efficient infection of the host. The toxin B has been shown to play an important role in *C. diffcile* pathogenesis in humans, since both, the A^+^B^+^ and A^−^B^+^ strains cause the same spectrum of clinical symptoms^39^. It has also been proposed that TcdB variant produced in the A^−^ strains is able to modify cellular Ras GTPases in the TcdA-specific manner^40^. These observations suggest that TcdB is sufficient for pathology in humans. In light of these, the increased level of toxin A observed in the case of the prophage-free strain CKH08 most likely does not compensate the significantly decreased production of toxin B (Fig. 9) which in turn results in lowering the cytopathic effect towards human colon cells (Fig. 7). This clearly indicates that the presence of phi027 prophage in the strain 500/12 is associated with the increased production of TcdB. The role of CDT in observed effects cannot be unambiguously confirmed, since levels of binary toxin were not checked in the study. Nevertheless, our preliminary analysis of transcriptomes of both, lysogenic and prophage-free strains suggested no changes in expression of genes *cdtA* and *cdtB* encoding this toxin (data not shown).

The molecular mechanism responsible for observed effects of the phi027 prophage on its host strain 500/12 most probably relies on the action of predicted transcriptional regulators encoded in the phage genome. There are 10 putative genes coding for transcription factors, including a predicted RNA polymerase sigma-70 subunit (*phiCDKH02_17*) and sigma factor-like heilx-turn-helix DNA-binding protein (*phiCDKH02_23*). Phage-encoded transcription factors have already been shown to influence major toxin production in *C. difficile*, as it is in the case of phage CD119 and its RepR protein downregulating expression of all genes in the PaLoc region^14^. Other phages, such as phiCD27 or phiCD38-2 also influence expression of toxin genes^16,17^, nevertheless in these cases no responsible mechanism has been elucidated.

Taken together, changes in the physiology of phi027 lysogenic *C. difficile* 500/12, along with altered interaction with colonic cells suggest better adaptation of such bacteria to their enteropathogenic lifestyle. This additionally emphasizes the important role of bacteriophages in shaping host-pathogen interactions.

## Materials and methods

### Bacterial strains and growth conditions

All the *E. coli* and *C. difficile* strains used in this study are listed in Table S2. The *DH5α E. coli* strain was used as the general host for plasmid construction and gene cloning. *E. coli* sExpress was used as the donor strain for the conjugation of *C. difficile*. The transformation of all the *E. coli* strains was conducted through rubidium chloride method for transformation competent *E. coli*. *E. coli* strains were grown in Luria-Bertani (LB) medium supplement with ampicillin (100 µg/mL), chloramphenicol (30 µg/mL), or kanamycin (50 µg/ mL) when necessary. *C. difficile* strains were cultivated in BHIS medium (Brain Heart Infusion, supplemented with 5 g/L yeast extract and 1 g/L L-cycloserine) at 37 °C, in an anaerobic chamber (BACTRON300, Sheldon Manufacturing, USA). BHIS medium was supplemented with the following antibiotics/inducer when appropriate: thiamphenicol (15 µg/mL), D-cycloserine (250 µg/mL), cefoxitin (10 µg/mL), lactose (40 mM).

The growth curves of *C. difficile* strains 500/12 and CKH08 were generated by taking OD_600 nm_ readings over an 8-hour period, with measurements recorded every two hours using measurement cuvettes. The BHI medium for C. difficile was pre-reduced before use. Bacterial cultures were maintained at 37 °C in an anaerobic chamber (BACTRON300, Sheldon Manufacturing, USA), and OD measurements were taken directly in the chamber using a spectrophotometer (SmartSpec Plus UV/Vis, Bio-Rad, USA). After 6 and 8 h of growth, the bacterial cultures in BHIS were diluted tenfold in the growth medium before taking measurements.

### Cell cultures

Three human epithelial cell lines were used in the study: HT-29 passaged 15–25 times prior to use (from the cell-line library at the Anaerobic Laboratory, Department of Medical Microbiology); HT-29 MTX passaged 5–15 times (continuous lines of human colorectal adenocarcinoma cells: non-mucosa, and mucosa, respectively) (European Collection of Authenticated Cell Cultures, ECACC, UK); and CCD 841 CoN passaged 5–15 times (continuous line of human normal colon epithelial cells; American Type Culture Collection, ATCC, USA). The cell lines were stored in the cell bank at -196 °C. Cells were cultured in Dulbecco’s Modified Eagle Medium (DMEM; Lonza, USA) with 4.5 g/L glucose and L-glutamine with addition of 10% heat-inactivated (56 °C, 30 min) fetal bovine serum (FBS) (Sigma-Aldrich, USA) and antibiotics: streptomycin, 100 µg/mL, penicillin, 100 U/mL, and amphotericin B, 250 µg/mL (Sigma-Aldrich, USA). Cells were thawed, replenished with culture fluid, and centrifuged at 1500×g for 5 min. Then, the growth medium was added to the cell pellet and transferred to 75-cm2 culture bottles (Corning, USA) and supplemented with DMEM culture fluid (Lonza, USA) and a relative air humidity of 95%. The supernatant medium was changed every three days.

To ensure the continuity of the cell culture and to obtain the appropriate number of cells for the study, the cell-line cultures were passaged when reaching a confluence of 90%. The medium above the growth surface was harvested and washed with phosphate buffer saline (PBS; Lonza, Switzerland), then the cells were trypsinized with EDTA (0.25%) (Sigma-Aldrich, USA) at 37 °C for 10 min. The trypsin effect was inactivated by adding 10 mL of fresh medium with 10% FBS. Then, the contents of the bottle were centrifuged at 1500×g for 5 min, after which the supernatant was removed, and 1 mL of medium was added to the remaining pellet. The cells were then counted with a Thoma chamber and diluted to the desired amount in a new flask with a fresh medium and 10% FBS. The excess cells were transferred to a new culture vessel. Cells intended for the experiment of bacterial adhesion to colon cells were cultured through the above method using 24-well plates in an incubator with a flow of 5% CO_2_ and a temperature of 37 °C. On the day before the experiment, the culture fluid was replaced with a new one, without the addition of antibiotics. Experiments were performed on mature cells, which were 15 days after seeding HT-29 and CCD 841 CoN cells and 21 days after seeding HT-29 MTX cells, all passaged 15–25 times^41^.

### Prophage induction and phage isolation

Prophage was identified, induced with mitomycin C, and isolated as described before^42^. Briefly, phage was induced by adding 3 µg/ml mitomycin C to the log-phase culture with further incubation at 37 °C for 12 h. The culture was then diluted 10-fold and phages were isolated using filtration of lysate through 0.22 μm filter. Host genomic DNA was removed from the lysate by treatment with 5 µg/ml of DNaseI at 37 °C for 1 h. The reaction was heat-inactivated at 99 °C for 10 min.

### Phage genome sequencing and annotation

Genomic DNA of phage induced from strain 500/12 was purified using a Phage DNA Isolation Kit (Norgen Biotek Corp., Thorold, Canada) following the manufacturer’s instructions. Whole-genome sequencing was performed by Genomed S.A. (Warsaw, Poland) on an Illumina MiSeq platform. High-quality paired-end reads were assembled *de novo* using SPAdes v. 3.13.0 (https://github.com/ablab/spades). The resulting consensus sequence was automatically annotated in the process of deposition in the GenBank database under the name phiCDKH02 and accession number PP767789.

### Whole-genome sequencing of clinical isolates

Genomic DNA of strains obtained from clinical samples was isolated using E.Z.N.A. Bacterial DNA Kit (Omega Bio-tek, USA). Whole-genome sequencings were performed on Illumina MiSeq platform, as described for the phage genome. Whole-genome sequencing of cured strain CKH08 and additional re-sequencing of strain 500/12 were performed by genXone S.A. (Suchy Las, Poland) on a GridION platform using Rapid Barcoding Kit v. 14 and FLO-MIN114 ver. 10.4.1 flowcell. In these cases reads were processed with MinKNOW v. 23.11.7, basecalling Dorado v. 7.2.13, Super-accurate basecalling model, assembled *de novo* with Flye v. 2.9.3, and polished with Medaka v. 1.11.1. Obtained scaffolds were automatically annotated in the process of deposition in the GenBank database. Corresponding accession numbers are listed in the Table S2.

### Identification of phiCDKH02 homologs

The consensus sequence of the phiCDKH02 was compared to the NCBI sequences database using online blastn suite of BLAST (https://blast.ncbi.nlm.nih.gov/Blast.cgi). Genomes found to contain full sequence of the prophage were selected for download. Pairwise alignment of phiCDKH02 genome was performed against the sequence of prophage phi027 present in the genome of strain R20219 (GenBank accession number FN545816) using progressive Mauve algorithm (Mauve v. 2.4.0)^43^.

### Analysis of sequenced genomes

Trimmed short reads were mapped against the genome of the strain R20219. Obtained consensus sequences were aligned using the progressive Mauve algorithm. A guide tree file generated by Mauve was used to draw a phylogenetic tree of analyzed genomes using FigTree v. 1.4.4 (https://github.com/rambaut/figtree/releases).

### In Silico ribotyping of phi027 lysogens

Sequences of genomes containing phi027 prophage (Table S4) were subjected to in silico ribotyping using in silico PCR Perl script (https://github.com/egonozer/in_silico_pcr) and ribotyping primers 5′-GTGCGGCTGGATCACCTCCT-3′ (16 S primer) and 5′-CCCTGCACCCTTAATAACTTGACC-3′ (23 S primer)^44^. Resulting hypothetical PCR products were formatted into a single data file along with results of in silico ribotyping of clinical isolates. The data file was read into the R package v. 4.2.1^45^ and subjected to hierarchical clustering based on the calculation of Euclidean distance. Output object was presented in the form of a tree to identify clusters of putative ribotypes.

### Building a maximum likelihood tree of phi027 lysogens

ORFs encoding proteins in the genome of the strain 500/12 were identified with tblastn^46^ querying ORFs of the *C. difficile* 630 (GenBank accession number CP010905). Resulting nucleotide sequences were stored in the single FASTA file. Next, it was used as a query in tblastn search of scaffolds obtained after the sequencing of genomes of clinical isolates and phi027 lysogens identified in the NCBI sequences database. Resulting files were parsed to identify proteins exhibiting the highest variability of amino acid sequence, as well as to calculate the average sequence change ratio expressed as a number of substitutions per 1000 amino acid residues. Proteins selected for this analysis are listed in Table (original *C. difficile* 630 annotation) Sequences were concatenated in a single FASTA file. The file was then used to perform the multiple sequence alignment in MEGA11 software^47^ and construct a maximum likelihood tree. To calculate the average ratio of nucleotide sequence change the 500/12 ORFs datafile was used as a query in blastn search of scaffolds obtained after sequencing of genomes of clinical isolates and phi027 lysogens identified in the NCBI sequences database. Resulting files were parsed to enable the calculation of this ratio expressed as a number of substitutions per 1000 nucleotides.

### Plasmids construction

All the plasmids used in this study are listed in Table S2. All the DNA primers used in this study are listed in Table S3. For the cloning purpose, PCR was performed using either Phusion High-Fidelity DNA Polymerase or Q5 High-Fidelity DNA Polymerase (NEB). DNA assembly was carried out using NEBuilder HiFi DNA Assembly Master Mix (NEB) following the manufacturer’s protocol. Details of plasmids construction are given in the Supporting Information.

### Conjugation of C. difficile and mutant screening

Plasmids were conjugated into *C. difficile* as described previously^48^ with some modifications. Briefly, 1 mL of the plasmid-harboring *E. coli* sExpress strain was centrifuged at 3000 g for 3 min and then washed once with sterilized LB medium. The cell pellet was transferred into the anaerobic chamber and mixed within 150 µL of an overnight culture of *C. difficile* grown in BHIS broth (with an OD600 of ∼0.8) previously incubated at 50 °C for 15 min to increase conjugation efficiency^49^. The mixture was spotted onto the BHIS agar plate (1.5%) with 20 µL per spot. The plate was then cultivated anaerobically at 37 °C for 10 h. Afterward, 1 mL of BHIS medium was added onto the plate surface to harvest cells. A 100 µL aliquot of the harvested cells was spread onto the BHIS-TDC agar plate, which contained thiamphenicol (15 µg/mL), D-cycloserine (250 µg/mL) and cefoxitin (10 µg/mL). Once visible, the colony was picked and inoculated into BHIS medium containing thiamphenicol (15 µg/mL) (BHIS-Tm). After cultivation for 12 h, the cell culture was serially diluted and 100 µL of each dilution was plated onto the BHISL-Tm plate, containing 15 µg/mL thiamphenicol and 40 mM lactose. After incubating for 36 h at 37 °C, colonies were picked randomly and screened for desirable mutants using colony PCR (cPCR). Specific gene-flanking diagnostic primers were used to verify desirable mutations. The mutations were further confirmed with Sanger sequencing.

### Plasmids curing

To cure the plasmid, the plasmid harboring mutant was inoculated into BHIS medium and subcultured (for about 10 times within 5 days). Then the culture was streaked on a BHIS agar plate. Colonies were carefully picked and dotted on both BHIS-Tm and BHIS agar plates (replica plating). The colonies susceptible to Tm were picked and propagated in 1 mL of BHIS medium. The desirable mutation within the clean mutant (the plasmid of which was cured) was further confirmed through PCR using primers ilvB-F, cotG-R, edit-F, int-R, phi027-F and phi027-R (Table S3).

### Testing the ability of *C. difficile* to produce biofilm in vitro

The biofilm assay was performed as described previously^50^ with some modifications. The wells of the 24 bottom microplate (Nunc, Roskilde, Denmark) have been replenished with 360 µL of BHI medium supplemented with 0.1 M of glucose. Afterward, wells were inoculated with 40 µL of overnight *C. difficile* culture incubated at 37 °C for 48 h under anaerobic conditions. Wells with BHI broth without inoculum were used as negative controls, while positive controls consisted of inoculated wells. After incubation, the liquid phase was aspired using a sterile pipette, and wells were washed twice with phosphate buffer saline (PBS) (Biomed, Kraków, Poland). Each well was then stained with crystal violet (CV) (Analab, Warsaw, Poland) for 10 min. The CV was removed, and the wells were washed eight times with PBS. After air-drying for 15 min at 37 °C, the CV within the biofilms was dissolved in ethanol and the absorbance was measured at 620 nm (A620) using a Multiskan SkyHigh Microplate Spectrophotometer (ThermoFisher Scientific, Singapore) microplate. All strains were tested three times. The mean values for each *C. difficile* strain were calculated.

### Analysis of *C. difficile* spore formation

Bacteria were plated on Columbia Agar containing 5% sheep blood and incubated for 48 h in an anaerobic chamber (Whitley A35 Workstation, UK). Grown colonies were inoculated in triplicates into the Ellners Broth (HiMedia Laboratories, India) modified as described by Duncan and Strong^51^. Cultures were cultivated in 37 °C at anaerobic conditions. The efficiency of sporulation was expressed as the percentage of sporulating cells within a population using modified method described by Åkerlund et al.^52^. At 24, 48 and 72 h timepoint samples of cultures were stained using Schaeffer-Fulton method^53^, and vegetative and sporulating cells were counted directly using a microscope without using a Bürker chamber. The percentage of sporulating cells was calculated once 100 vegetative cells have been counted. Results were evaluated for statistical significance using one-way ANOVA with Tukey’s *post hoc* test.

### Adhesion of *C. difficile* strains to human epithelial cell lines

The method was described by Piotrowski et al. with some modifications^54^. For the experiment, all cell lines were prepared as described above. Sub-confluent cells in 24-well plate were washed twice using PBS, then 400 µl fresh pre-warmed (37 °C) medium without antibiotic/antimycotic solution. Prepared plates were incubated for 4 h under cell culture conditions. Bacterial inoculum was prepared by dissolving single colonies of *C. difficile* strains grown on Columbia agar with 5% sheep blood grown for 24 h and adjusting to McFarland standard of 3.0 in suspension medium (bioM´erieux, France)0.100 µl of inoculum was added to each well and incubated for two hours. After incubation media were aspirated and wells washed twice with PBS. The cells were trypsinized for 10 min at 37 °C and 500 µl of fresh medium with 10% FBS was added to deactivate trypsin. The content of each well was transferred to sterile Eppendorf and diluted 10 times using PBS. Then 20 µl of dilution was used to inoculate corresponding Columbia Agar with 5% sheep blood plate and incubated for 48 h at 37 °C under anaerobic conditions. Every dilution was seeded in duplicate and the test was performed three times. Grown colonies were counted and averaged, percentage of adhesion was calculated using the formula: adhesion (%) = 100 x bacterial count in the sample / bacterial count in control, where control represents 100% adhesion^41^. Results were evaluated for statistical significance using one-way ANOVA with Tukey’s *post hoc* test.

### Cytotoxicity assays

The cytotoxic effect of *C. difficile* culture supernatants was performed using the cytopathic effect method (CPE)^55^. HT29 (passage 34) and CCD841 CoN (passage 18) cells were seeded into the 24-well plates and grown until they formed a monolayer. Single colonies of *C. difficile* strains were inoculated in BHI liquid medium and cultured for 4 days under anaerobic conditions. Samples of the cultures from the bottom of the test tubes were transferred into the Eppendorf tubes and centrifuged for 3.5 min at 800 x g. Supernatants were serially diluted in fresh cell culture media without FBS and antibiotics. 250 µl of each dilution was added to wells containing cells and plates were incubated for 24 and 48 h at 37 °C with 5% CO_2_. The cytotoxic effect was considered on the wells in which 50% of cells were exhibiting the cytopathy. Control of the test was performed using toxin neutralization with antitoxin B (*C. difficile* Tox-B Test, TechLab, USA) added to the cells treated with 50 µl of supernatant. Cells were observed using the Eclipse Ts2 inverted microscope (Nikon Corporation, Japan).

### Measurement of clostridial toxin concentration

The assessment of toxin A (TcdA) and toxin B (TcdB) levels in the strains was carried out using the ELISA immunoassay, according to the manufacturer’s instructions (tgcBIOMICS, Germany), with a sensitivity of TcdA/TcdB 0.5 ng/ml. *Clostridioides difficile* strains 500/12 and CKH08 were plated from the microbank (Pro-Lab Diagnostics, Canada) onto Columbia Agar with 5% sheep blood. The plates were incubated in anaerobic conditions for 24 h, followed by a transfer of the culture and further incubation for 48 h in anaerobic conditions (Whitley A35 Workstation, UK). Twenty colonies of each strain were collected, suspended in 2 ml of diluent buffer, and centrifuged at 2500G for 5 min. Further assays were performed on the collected supernatant according to a two-step protocol. The measurements were carried out in eight repetitions. Absorbance was read at a wavelength of 450/620 nm (uQuant Bio-Tek, US).

## Electronic supplementary material

Below is the link to the electronic supplementary material.

Supplementary Material 1

Supplementary Material 2

Supplementary Material 3

Supplementary Material 4

Supplementary Material 5

Supplementary Material 6

Supplementary Material 7

Supplementary Material 8

Supplementary Material 9

Supplementary Material 10

Supplementary Material 11

## Data Availability

The genomic sequences of the phiCDKH02 phage, and the 500/12 and CKH08 strains were deposited in GenBank with accession numbers PP767789, CP180209 and CP180211 , respectively. The supporting data is provided in the supplementary information files, including the plasmid construction, Fig S1, Fig S2,Fig S3,Table S1, Table S2, Table S3, and Table S4.
