# Supplementary material for: The phi027 bacteriophage influences physiology and virulence of the lysogenic strain of Clostridioides difficile
Source: Sci Rep. 2025 May 29;15:18856. doi: 10.1038/s41598-025-04106-0 (PMC12122855; doi:10.1038/s41598-025-04106-0)
Supplement: Supplementary file 1 — Supplementary Material 1 [file 41598_2025_4106_MOESM1_ESM.docx]

**A Python-Based Algorithm to Design Primers for One-Step-Assembly (OSA).** A Python-based algorithm, denoted OSA Primer Finder (OPF), was used to search potential protospacers in DNA sequences flanking the phiCDKH02/*phi027* locus and design corresponding primers for constructing the plasmid through OSA. OPF is run with Python (version 3.11; https://www. python.org/). Detailed procedures for using OPF to design primers are described previously (Hong et.al, 2018).

**Plasmid construction**

To construct pEcCdH01, the small RNA promoter (sRNAP; promoter of sCbei_5830) from *C. beijerinckii* 8052 genome with the crRNA sequence (5’-TAATTTCTACTCTTGTAGATCATC TGATAAGAAGGACTTAATA-3’) were synthesized by GenScript and introduced into pUC19, generating pEcCdH01.

The plasmid pEcCdH07 was designed to delete the ~55.9-kb *phiCDKH02/phi027* locus (JBCJLD010000007.1, 93652-149601). The fragment sRNAP::crRNA-*phiCDKH02*, containing the specific spacer 5’- GCCACCCATTTGCCACCGTATTA-3’ (PAM sequence: 5’-TTTT-3’), was generated with primers YW3105/phi500_S1_0. Homology arms *phiCDKH02*-Up-arm and *phiCDKH02*-Down-arm were generated with primers phi500_S1_1/phi500_S1_2   and phi500_S1_1/phi500_S1_2, respectively. Then the three fragments were assembled with BtgZI-linearized pWH34 to generate pEcCdH07. The plasmid pEcCdH08 was also designed to delete the ~55.9-kb *phiCDKH02/phi027* locus, however, with two spacers (5’- GCCACCCATTTGCCACCGTATTA-3’ and 5’-TCAGCAACACCATTTGCTAAGTC-3’) in order to improve DSB efficiency for this extremely large gene deletion. The fragment sRNAP::crRNA-*phiCDKH02 double* was generated with primers YW3105/phi500_S1S2_0, in which a second spacer 5’-TCAGCAACACCATTTGCTAAGTC-3’ was added to the 3’-end of sRNAP::crRNA-*phiCD630-2*. Homology arms *phiCDKH02*-Up-arm and *phiCDKH02*-Down-arm were generated with primers phi500_S1S2_1/phi500_S1_2 and phi500_S1_1/phi500_S1_2, respectively. The rest of the plasmid construction process was similar to the construction of pEcCdH07.
