# Supplementary material for: The phi027 bacteriophage influences physiology and virulence of the lysogenic strain of Clostridioides difficile
Source: Sci Rep. 2025 May 29;15:18856. doi: 10.1038/s41598-025-04106-0 (PMC12122855; doi:10.1038/s41598-025-04106-0)
Supplement: Supplementary file 2 — Supplementary Material 2 [file 41598_2025_4106_MOESM2_ESM.docx]

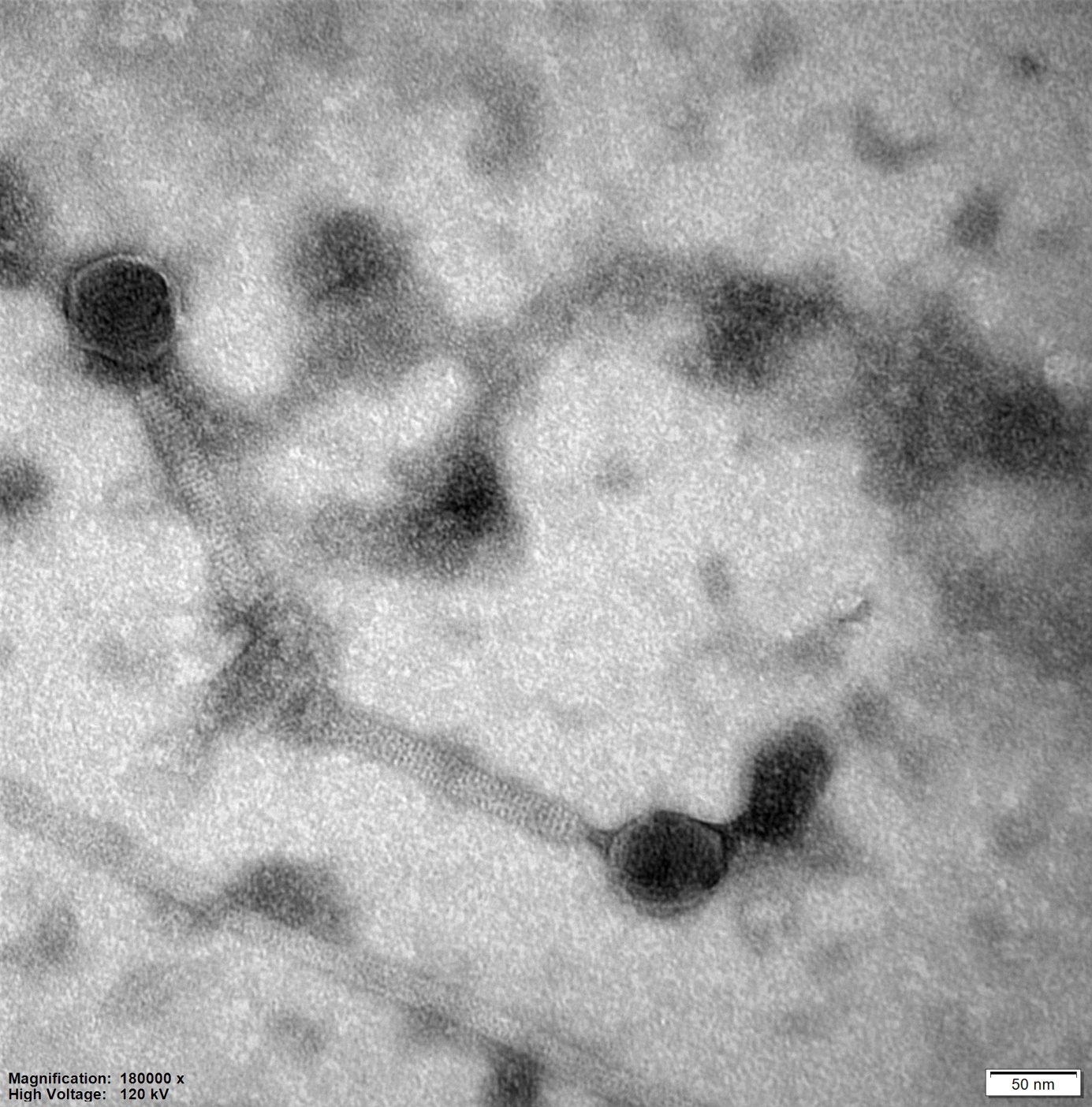


**Fig. S1** phiCDKH02 morphology observed using a transmission electron microscope Tecnai Spirit BioTWIN microscope (FEI) at an acceleration voltage of 120 kV. Phage particles were stained with 2% uranyl acetate.
