# Supplementary material for: The phi027 bacteriophage influences physiology and virulence of the lysogenic strain of Clostridioides difficile
Source: Sci Rep. 2025 May 29;15:18856. doi: 10.1038/s41598-025-04106-0 (PMC12122855; doi:10.1038/s41598-025-04106-0)
Supplement: Supplementary file 3 — Supplementary Material 3 [file 41598_2025_4106_MOESM3_ESM.docx]

**Fig. S3** Biofilm formation by 500/12 and CKH08 as assessed by CV staining. Error bars represent standard deviation calculated for three repeats. Student’s t test *p*-value calculated for observed differences in means equals 0.3585.
