# Supplementary material for: The phi027 bacteriophage influences physiology and virulence of the lysogenic strain of Clostridioides difficile
Source: Sci Rep. 2025 May 29;15:18856. doi: 10.1038/s41598-025-04106-0 (PMC12122855; doi:10.1038/s41598-025-04106-0)
Supplement: Supplementary file 4 — Supplementary Material 4 [file 41598_2025_4106_MOESM4_ESM.docx]

**Table S1**. ORFs locations, transcriptional orientations, sizes of encoded proteins, possible function, BLAST hit, alignment and E value

| **Locus tag** | **Coding region (bp)** | **Strand** | **Protein size （aa）** | **Predicted functional** | **BLAST hit [source organism]^a^** | **Accession number** | **Alignment**  **(%ID)** | **E value** |
| --- | --- | --- | --- | --- | --- | --- | --- | --- |
|  |  |  |  |  |  |  |  |  |
| phiCDKH02 _01 | 206-1336 | - | 376 | integrase | site-specific integrase [*Clostridioides difficile*] | WP_003429803.1 | 376/376 (100%) | 0.0 |
| phiCDKH02_02 | 1415-2122 | - | 235 | DNA-binding domain protein | HIRAN domain-containing protein [*Clostridioides difficile*] | WP_009888800.1 | 235/235  (100%) | 0.0 |
| phiCDKH02_03 | 2135-3004 | - | 289 | HipA toxin  (a type II toxin-antitoxin (TA) system) | HipA domain-containing protein [*Clostridioides difficile*] | WP_009888801.1 | 289/289  (100%) | 0.0 |
| phiCDKH02_04 | 3624 -4130 | - | 168 | metallopeptidase | ImmA/IrrE family metallo-endopeptidase [*Clostridioides difficile*] | WP_003429804.1 | 168/168  (100%) | 2e-114 |
| phiCDKH02_05 | 4206 - 4754 | - | 182 | transcriptional factor | helix-turn-helix transcriptional regulator [*Clostridioides difficile*] | WP_009893126.1 | 182/182  (100%) | 5e-124 |
| phiCDKH02_06 | 4766- 5107 | - | 113 | transcriptional factor | XRE family transcriptional regulator [*Clostridioides difficile*] | WP_009888806.1 | 113/113 (100%) | 2e-77 |
| phiCDKH02_07 | 5284-5487 | + | 67 | transcriptional factor | [helix-turn-helix domain-containing protein [*Clostridioides difficile*]](https://blast.ncbi.nlm.nih.gov/Blast.cgi#alnHdr_WP_009893129) | [WP_009893129.1](https://www.ncbi.nlm.nih.gov/protein/WP_009893129.1?report=genbank&log$=prottop&blast_rank=1&RID=AZX06J0U016) | 67/67 (100%) | 8e-39 |
| phiCDKH02_08 | 5535-6344 | + | 269 | phage antirepressor | phage antirepressor [*Clostridioides difficile*] | WP_009888810.1 | 269/269 (100%) | 0.0 |
| phiCDKH02_09 | 6391-6600 | + | 69 | hypothetical protein | hypothetical protein [*Clostridioides difficile*] | WP_009888812.1 | 69/69  (100%) | 8e-41 |
| phiCDKH02_10 | 6610-6984 | - | 124 | unknown function | DUF6173 family protein [*Clostridioides difficile*] | [WP_003433505.1](https://www.ncbi.nlm.nih.gov/protein/WP_003433505.1?report=genbank&log$=prottop&blast_rank=1&RID=AZYAWM4D013) | 124/124 (100%) | 2e-85 |
| phiCDKH02_11 | 7060-7257 | + | 65 | transcriptional factor | helix-turn-helix transcriptional regulator [*Clostridioides difficile*] | [WP_009888813.1](https://www.ncbi.nlm.nih.gov/protein/WP_009888813.1?report=genbank&log$=prottop&blast_rank=1&RID=B247BZGX013) | 65/65 (100%) | 2e-36 |
| phiCDKH02_12 | 7288-7680 | + | 130 | transcriptional factor | [helix-turn-helix domain-containing protein [*Clostridioides difficile*]](https://blast.ncbi.nlm.nih.gov/Blast.cgi#alnHdr_WP_009888815) | [WP_009888815.1](https://www.ncbi.nlm.nih.gov/protein/WP_009888815.1?report=genbank&log$=prottop&blast_rank=1&RID=B24KWW0T013) | 130/130  (100%) | 1e-85 |
| phiCDKH02_13 | 7667-7810 | + | 47 | hypothetical protein | hypothetical protein [*Clostridioides difficile*] | [WP_009888817.1](https://www.ncbi.nlm.nih.gov/protein/WP_009888817.1?report=genbank&log$=prottop&blast_rank=1&RID=B24X5HE5013) | 47/47 (100%) | 3e-20 |
| phiCDKH02_14 | 7881-8318 | + | 145 | hypothetical protein | hypothetical protein [*Clostridioides difficile*] | [WP_003433513.1](https://www.ncbi.nlm.nih.gov/protein/WP_003433513.1?report=genbank&log$=prottop&blast_rank=1&RID=B25CW7BG01N) | 145/145  (100%) | 1e-91 |
| phiCDKH02_15 | 8330-9496 | + | 388 | unknown function | DUF2800 domain-containing protein [*Clostridioides difficile*] | [WP_009888818.1](https://www.ncbi.nlm.nih.gov/protein/WP_009888818.1?report=genbank&log$=prottop&blast_rank=1&RID=B25SYA70013) | 388/388 (100%) | 0.0 |
| phiCDKH02_16 | 9511-9846 | + | 111 | hypothetical protein | hypothetical protein [*Clostridioides difficile*] | [WP_009888819.1](https://www.ncbi.nlm.nih.gov/protein/WP_009888819.1?report=genbank&log$=prottop&blast_rank=1&RID=B27AKUXD013) | 111/111 (100%) | 3e-71 |
| phiCDKH02_17 | 9899-10471 | + | 190 | RNA polymerase subunit sigma -70 | RNA polymerase subunit sigma -70 [*Clostridioides difficile*] | [WP_003429822.1](https://www.ncbi.nlm.nih.gov/protein/WP_003429822.1?report=genbank&log$=prottop&blast_rank=1&RID=B27NHSBN013) | 190/190  (100%) | 6e-135 |
| phiCDKH02_18 | 10494-11066 | + | 190 | unknown function | [DUF2815 family protein [*Clostridioides difficile*]](https://blast.ncbi.nlm.nih.gov/Blast.cgi#alnHdr_WP_003433516) | [WP_003433516.1](https://www.ncbi.nlm.nih.gov/protein/WP_003433516.1?report=genbank&log$=prottop&blast_rank=1&RID=B2832Z3R016) | 190/190 (100%) | 2e-136 |
| phiCDKH02_19 | 11066-13024 | + | 652 | DNA polymerase | DNA polymerase [*Clostridioides difficile*] | WP_009888821.1 | 652/652 (100%) | 0.0 |
| phiCDKH02_20 | 13036-15453 | + | 805 | virulence-associated E protein | virulence-associated E family protein [*Clostridioides difficile*] | WP_009888822.1 | 805/805 (100%) | 0.0 |
| phiCDKH02_21 | 16091-17380 | + | 429 | helicase | DEAD/DEAH box helicase family protein [*Clostridioides difficile F253*] | [EQI11293.1](https://www.ncbi.nlm.nih.gov/protein/EQI11293.1?report=genbank&log$=prottop&blast_rank=1&RID=B2C5NMUA013) | 429/429 (100%) | **0.0** |
| phiCDKH02_22 | 17404-17541 | + | 45 | hypothetical protein | hypothetical protein [*Clostridioides difficile*] | [WP_009888824.1](https://www.ncbi.nlm.nih.gov/protein/WP_009888824.1?report=genbank&log$=prottop&blast_rank=1&RID=B2CGKVDR016) | 45/45 (100%) | 6e-23 |
| phiCDKH02_23 | 17534-18013 | + | 159 | transcriptional factor | s[igma factor-like helix-turn-helix DNA-binding protein [*Clostridioides difficile*]](https://blast.ncbi.nlm.nih.gov/Blast.cgi#alnHdr_WP_009888825) | [WP_009888825.1](https://www.ncbi.nlm.nih.gov/protein/WP_009888825.1?report=genbank&log$=prottop&blast_rank=1&RID=B9MFYX1H013) | 159/159 (100%) | 2e-108 |
| phiCDKH02_24 | 18296-18748 | + | 150 | phage antirepressor | [phage antirepressor [*Clostridioides difficile*]](https://blast.ncbi.nlm.nih.gov/Blast.cgi#alnHdr_WP_009888826) | [WP_009888826.1](https://www.ncbi.nlm.nih.gov/protein/WP_009888826.1?report=genbank&log$=prottop&blast_rank=1&RID=B9NVA03H016) | 150/150 (100%) | 1e-104 |
| phiCDKH02_25 | 19040-20014 | + | 324 | hypothetical protein | hypothetical protein [*Clostridioides difficile*] | [WP_009888827.1](https://www.ncbi.nlm.nih.gov/protein/WP_009888827.1?report=genbank&log$=prottop&blast_rank=1&RID=B9P4XYFX013) | 324/324  (100%) | 0.0 |
| phiCDKH02_26 | 20035-20544 | + | 169 | hypothetical protein | hypothetical protein [*Clostridioides difficile*] | [WP_003429834.1](https://www.ncbi.nlm.nih.gov/protein/WP_003429834.1?report=genbank&log$=prottop&blast_rank=1&RID=B9PADHS1016) | 169/169  (100%) | 6e-114 |
| phiCDKH02_27 | 20614-21306 | + | 230 | p[hage terminase small subunit](https://blast.ncbi.nlm.nih.gov/Blast.cgi#alnHdr_WP_009888829) | p[hage terminase small subunit [*Clostridioides difficile*]](https://blast.ncbi.nlm.nih.gov/Blast.cgi#alnHdr_WP_009888829) | [WP_009888829.1](https://www.ncbi.nlm.nih.gov/protein/WP_009888829.1?report=genbank&log$=prottop&blast_rank=1&RID=B9PNB3Z8016) | 230/230  (100%) | 1e-165 |
| phiCDKH02_28 | 21296-22537 | + | 413 | phage terminase large subunit | [BSX family phage terminase large subunit [*Clostridioides difficile*]](https://blast.ncbi.nlm.nih.gov/Blast.cgi#alnHdr_WP_012816148) | [WP_012816148.1](https://www.ncbi.nlm.nih.gov/protein/WP_012816148.1?report=genbank&log$=prottop&blast_rank=1&RID=B9R0J2CU013) | 413/413  (100%) | 0.0 |
| phiCDKH02_29 | 22543-23985 | + | 480 | phage portal protein | [phage portal protein [*Clostridioides difficile*]](https://blast.ncbi.nlm.nih.gov/Blast.cgi#alnHdr_WP_009888831) | [WP_009888831.1](https://www.ncbi.nlm.nih.gov/protein/WP_009888831.1?report=genbank&log$=prottop&blast_rank=1&RID=B9R7DRAX016) | 480/480 (100%) | 0.0 |
| phiCDKH02_30 | 23975-25510 | + | 511 | minor capsid protein | [minor capsid protein [*Clostridioides difficile*]](https://blast.ncbi.nlm.nih.gov/Blast.cgi#alnHdr_WP_009893134) | [WP_009893134.1](https://www.ncbi.nlm.nih.gov/protein/WP_009893134.1?report=genbank&log$=prottop&blast_rank=1&RID=B9RPSGYU013) | 511/511 (100%) | 0.0 |
| phiCDKH02_31 | 25512-25760 | + | 82 | hypothetical protein | hypothetical protein [*Clostridioides difficile*] | [WP_284971294.1](https://www.ncbi.nlm.nih.gov/protein/WP_284971294.1?report=genbank&log$=prottop&blast_rank=1&RID=B9RZTVY301N) | 82/82 (100%) | 4e-50 |
| phiCDKH02_32 | 25828-26391 | + | 187 | scaffolding protein | phage scaffolding protein [*Clostridioides difficile*] | WP_009888833.1 | 187/187  (100%) | 1e-131 |
| phiCDKH02_33 | 26403-27293 | + | 296 | capsid protein | [capsid protein [*Clostridioides difficile*]](https://blast.ncbi.nlm.nih.gov/Blast.cgi#alnHdr_MDO0248834) | [MDO0248834.1](https://www.ncbi.nlm.nih.gov/protein/MDO0248834.1?report=genbank&log$=prottop&blast_rank=1&RID=BW9Y8NGN016) | 296/296  (100%) | 0.0 |
| phiCDKH02_34 | 27312-27545 | + | 77 | hypothetical protein | hypothetical protein [*Clostridioides difficile*] | [WP_009888835.1](https://www.ncbi.nlm.nih.gov/protein/WP_009888835.1?report=genbank&log$=prottop&blast_rank=1&RID=BWA70EUV016) | 77/77 (100%) | 2e-45 |
| phiCDKH02_35 | 27555-27956 | + | 133 | hypothetical protein | [hypothetical protein HMPREF1123_03668 [*Clostridioides difficile* 050-P50-2011]](https://blast.ncbi.nlm.nih.gov/Blast.cgi#alnHdr_EHJ25479) | [EHJ25479.1](https://www.ncbi.nlm.nih.gov/protein/EHJ25479.1?report=genbank&log$=prottop&blast_rank=1&RID=BWAD5UYE016) | 133/133 (100%) | 5e-90 |
| phiCDKH02_36 | 27950-28297 | + | 115 | unknown function | [Putative phage protein [*Clostridioides difficile* E13]](https://blast.ncbi.nlm.nih.gov/Blast.cgi#alnHdr_CCL05056) | [CCL05056.1](https://www.ncbi.nlm.nih.gov/protein/CCL05056.1?report=genbank&log$=prottop&blast_rank=1&RID=BWAMT83D016) | 115/115 (100%) | 8e-77 |
| phiCDKH02_37 | 28297-28587 | + | 96 | tail-component protein | [HK97 gp10 family phage protein [*Clostridioides*]](https://blast.ncbi.nlm.nih.gov/Blast.cgi#alnHdr_WP_009893135) | [WP_009893135.1](https://www.ncbi.nlm.nih.gov/protein/WP_009893135.1?report=genbank&log$=prottop&blast_rank=1&RID=BWAVD6ZM01N) | 96/96 (100%) | 2e-64 |
| phiCDKH02_38 | 28697-29503 | + | 268 | hypothetical protein | [hypothetical protein QQS_0957 [*Clostridioides difficile* P6]](https://blast.ncbi.nlm.nih.gov/Blast.cgi#alnHdr_EQJ03299) | [EQJ03299.1](https://www.ncbi.nlm.nih.gov/protein/EQJ03299.1?report=genbank&log$=prottop&blast_rank=1&RID=BWEG7UFA01N) | 268/268  (100%) | 0.0 |
| phiCDKH02_39 | 29852-30289 | + | 145 | unknown function | D[UF6838 family protein [Clostridioides difficile]](https://blast.ncbi.nlm.nih.gov/Blast.cgi#alnHdr_WP_009888839) | [WP_009888839.1](https://www.ncbi.nlm.nih.gov/protein/WP_009888839.1?report=genbank&log$=prottop&blast_rank=1&RID=BWGSXWUU01N) | 145/145  (100%) | 5e-99 |
| phiCDKH02_40 | 30282-30458 | + | 58 | hypothetical protein | hypothetical protein [*Clostridioides*] | [WP_009888840.1](https://www.ncbi.nlm.nih.gov/protein/WP_009888840.1?report=genbank&log$=prottop&blast_rank=1&RID=BWH1EY13013) | 58/58 (100%) | 1e-31 |
| phiCDKH02_41 | 30459-31769 | + | 436 | phage tail sheath protein | phage tail sheath family protein [*Clostridioides difficile*] | [WP_009893136.1](https://www.ncbi.nlm.nih.gov/protein/WP_009893136.1?report=genbank&log$=prottop&blast_rank=1&RID=BWHKW4MS016) | 436/436 (98.28%) | 0.0 |
| phiCDKH02_42 | 31786-32256 | + | 156 | phage tail tube protein | phage tail tube protein [*Clostridioides*] | [WP_009888842.1](https://www.ncbi.nlm.nih.gov/protein/WP_009888842.1?report=genbank&log$=prottop&blast_rank=1&RID=C4JSK32M013) | 156/156 (100%) | 2e-111 |
| phiCDKH02_ 43 | 32315-33142 | + | 131 | deoxyuridine 5'-triphosphate nucleotidohydrolase | Phage deoxyuridine 5'-triphosphate nucleotidohydrolase [*Clostridioides difficile*] | [VFC12109.1](https://www.ncbi.nlm.nih.gov/protein/VFC12109.1?report=genbank&log$=prottop&blast_rank=16&RID=C4K7HDK0013) | 131/131 (100%) | 6e-88 |
| phiCDKH02_44 | 33214-33654 | + | 146 | phage portal protein | phage portal protein [*Clostridioides difficile*] | [HBG0430505.1](https://www.ncbi.nlm.nih.gov/protein/HBG0430505.1?report=genbank&log$=prottop&blast_rank=1&RID=C4KRUXUS016) | 146/146 (100%) | 2e-102 |
| phiCDKH02_45 | 33729-33848 | + | 39 | unknown function | putative unknown function protein [*Peptoclostridium* phage p630P1] [*Clostridioides difficile* 630] | [AJP10638.2](https://www.ncbi.nlm.nih.gov/protein/AJP10638.2?report=genbank&log$=prottop&blast_rank=13&RID=C4KXEPTR016) | 39/39 (100%) | 3e-15 |
| phiCDKH02_46 | 34503-34607 | + | 34 | unknown function | hypothetical phage protein CDIF630_01087 [*Peptoclostridium phage* p630P1] [*Clostridioides difficile* 630] | [AJP10639.1](https://www.ncbi.nlm.nih.gov/protein/AJP10639.1?report=genbank&log$=prottop&blast_rank=1&RID=C4M5TC4X013) | 34/34 (100%) | 2e-14 |
| phiCDKH02_47 | 35493-35681 | + | 62 | hypothetical protein | hypothetical protein [*Clostridioides difficile*] | WP_003429858.1 | 62/62 (100%) | 9e-34 |
| phiCDKH02_48 | 35800-36666 | + | 288 | transcriptional factor | BRO family protein [*Clostridioides difficile*] | [WP_009888845.1](https://www.ncbi.nlm.nih.gov/protein/WP_009888845.1?report=genbank&log$=prottop&blast_rank=1&RID=C4NMP0UR016) | 288/288  (100%) | 0.0 |
| phiCDKH02_49 | 36719-36853 | + | 44 | hypothetical protein | hypothetical protein [*Clostridioides difficile*] | [WP_142797203.1](https://www.ncbi.nlm.nih.gov/protein/WP_142797203.1?report=genbank&log$=prottop&blast_rank=1&RID=C4P1Z8RC013) | 44/44 (100%) | 3e-20 |
| phiCDKH02_50 | 37531-38058 | + | 175 | lipoprotein | [DUF4352 domain-containing protein [*Clostridioides difficile*]](https://blast.ncbi.nlm.nih.gov/Blast.cgi#alnHdr_WP_009888847) | [WP_009888847.1](https://www.ncbi.nlm.nih.gov/protein/WP_009888847.1?report=genbank&log$=prottop&blast_rank=1&RID=C4P8UM66013) | 175/175 (100%) | 1e-122 |
| phiCDKH02_51 | 38196-38963 | + | 255 | transcriptional factor | [DUF4428 domain-containing protein [*Clostridioides difficile*]](https://blast.ncbi.nlm.nih.gov/Blast.cgi#alnHdr_WP_009888848) | [WP_009888848.1](https://www.ncbi.nlm.nih.gov/protein/WP_009888848.1?report=genbank&log$=prottop&blast_rank=1&RID=C4PNWP5P013) | 255/255 (100%) | 3e-180 |
| phiCDKH02_52 | 39029-41401 | + | 790 | tape measure protein | [tape measure protein [*Clostridioides difficile*]](https://blast.ncbi.nlm.nih.gov/Blast.cgi#alnHdr_WP_009893138) | [WP_009893138.1](https://www.ncbi.nlm.nih.gov/protein/WP_009893138.1?report=genbank&log$=prottop&blast_rank=1&RID=C4RHTWP5013) | 790/790 (100%) | 0.0 |
| phiCDKH02_53 | 41418-42107 | + | 229 | peptidoglycan hydrolase | [LysM peptidoglycan-binding domain-containing protein [*Clostridioides difficile*]](https://blast.ncbi.nlm.nih.gov/Blast.cgi#alnHdr_WP_009888849) | [WP_009888849.1](https://www.ncbi.nlm.nih.gov/protein/WP_009888849.1?report=genbank&log$=prottop&blast_rank=1&RID=C4RSUTKM013) | 229/229 (100%) | 1e-165 |
| phiCDKH02_54 | 42100-43992 | + | 630 | endopeptidase | NlpC/P60 family protein [*Clostridioides difficile*] | [WP_009893140.1](https://www.ncbi.nlm.nih.gov/protein/WP_009893140.1?report=genbank&log$=prottop&blast_rank=1&RID=C4S43KNT016) | 630/630  (100%) | 0.0 |
| phiCDKH02_55 | 44006-44266 | + | 86 | unknown function | DUF2577 domain-containing protein [*Clostridioides difficile*] | [WP_009893142.1](https://www.ncbi.nlm.nih.gov/protein/WP_009893142.1?report=genbank&log$=prottop&blast_rank=1&RID=C4U6S40N01N) | 86/86 (100%) | 7e-54 |
| phiCDKH02_56 | 44271-44690 | + | 139 | unknown function | DUF2634 domain-containing protein [*Clostridioides difficile*] | [WP_009888851.1](https://www.ncbi.nlm.nih.gov/protein/WP_009888851.1?report=genbank&log$=prottop&blast_rank=1&RID=C4UHSY7U013) | 139/139 (100%) | 3e-95 |
| phiCDKH02_57 | 44691-45740 | + | 349 | baseplate protein | baseplate J/gp47 family protein [*Clostridioides difficile*] | [WP_009888852.1](https://www.ncbi.nlm.nih.gov/protein/WP_009888852.1?report=genbank&log$=prottop&blast_rank=1&RID=C4V48V90016) | 349/349 (100%) | 0.0 |
| phiCDKH02_58 | 45733-46350 | + | 205 | unknown function | [YmfQ family protein [Clostridioides difficile]](https://blast.ncbi.nlm.nih.gov/Blast.cgi#alnHdr_WP_003433579) | [WP_003433579.1](https://www.ncbi.nlm.nih.gov/protein/WP_003433579.1?report=genbank&log$=prottop&blast_rank=1&RID=C4VDK8DG013) | 205/205 (100%) | 9e-148 |
| phiCDKH02_59 | 46362-47387 | + | 341 | phage tail protein | [phage tail protein [Clostridioides difficile]](https://blast.ncbi.nlm.nih.gov/Blast.cgi#alnHdr_WP_009888853) | [WP_009888853.1](https://www.ncbi.nlm.nih.gov/protein/WP_009888853.1?report=genbank&log$=prottop&blast_rank=1&RID=C4W2JN4X013) | 341/341 (100%) | 0.0 |
| phiCDKH02_60 | 47404-48930 | + | 508 | phage tail fiber protein | Phage tail fiber protein [Clostridioides difficile] | [WP_009893144.1](https://www.ncbi.nlm.nih.gov/protein/WP_009893144.1?report=genbank&log$=prottop&blast_rank=1&RID=C4W9XA52013) | 508/508 (100%) | 0.0 |
| phiCDKH02_61 | 48945-49238 | + | 97 | hypothetical protein | hypothetical protein [Clostridioides difficile] | [WP_009889429.1](https://www.ncbi.nlm.nih.gov/protein/WP_009889429.1?report=genbank&log$=prottop&blast_rank=1&RID=C6M6386C013) | 97/97 (100%) | 1e-61 |
| phiCDKH02_62 | 49238-49420 | + | 60 | hypothetical protein | hypothetical protein [Clostridioides difficile] | [WP_009889431.1](https://www.ncbi.nlm.nih.gov/protein/WP_009889431.1?report=genbank&log$=prottop&blast_rank=1&RID=C6MMVYVG013) | 60/60 (100%) | 5e-34 |
| phiCDKH02_63 | 49454-50020 | + | 188 | hypothetical protein | hypothetical protein [Clostridioides difficile] | [WP_009889434.1](https://www.ncbi.nlm.nih.gov/protein/WP_009889434.1?report=genbank&log$=prottop&blast_rank=1&RID=C6N0DSS9016) | 188/188 (100%) | 3e-134 |
| phiCDKH02_64 | 50051-50341 | + | 96 | hypothetical protein | hypothetical protein [Clostridioides difficile] | [WP_009889435.1](https://www.ncbi.nlm.nih.gov/protein/WP_009889435.1?report=genbank&log$=prottop&blast_rank=1&RID=C6PD3A4W01N) | 96/96 (100%) | 2e-62 |
| phiCDKH02_65 | 50345-50602 | + | 85 | holin | p[hage holin family protein [*Clostridioides difficile*]](https://blast.ncbi.nlm.nih.gov/Blast.cgi#alnHdr_WP_009889440) | [WP_009889440.1](https://www.ncbi.nlm.nih.gov/protein/WP_009889440.1?report=genbank&log$=prottop&blast_rank=1&RID=C6PMDECP01N) | 85/85 (100%) | 1e-51 |
| phiCDKH02_66 | 50863-51726 | + | 287 | membrane-bound metalloproteases | [Abi family protein [*Clostridioides difficile*]](https://blast.ncbi.nlm.nih.gov/Blast.cgi#alnHdr_WP_003429958) | [WP_003429958.1](https://www.ncbi.nlm.nih.gov/protein/WP_003429958.1?report=genbank&log$=prottop&blast_rank=1&RID=C6RRKFHY016) | 287/287 (100%) | 0.0 |
| phiCDKH02_67 | 51805-52617 | + | 270 | peptidoglycan hydrolase | [N-acetylmuramoyl-L-alanine amidase [*Clostridioides difficile*]](https://blast.ncbi.nlm.nih.gov/Blast.cgi#alnHdr_WP_009889444) | [WP_009889444.1](https://www.ncbi.nlm.nih.gov/protein/WP_009889444.1?report=genbank&log$=prottop&blast_rank=1&RID=C6U5BC8C013) | 270/270 (100%) | 0.0 |
|  |  |  |  |  |  |  |  |  |
| phiCDKH02_68 | 52771-55110 | + | 779 | cell wall-binding repeat-containing protein | [cell wall-binding repeat-containing protein [Clostridioides difficile]](https://blast.ncbi.nlm.nih.gov/Blast.cgi#alnHdr_WP_009893147) | [WP_009893147.1](https://www.ncbi.nlm.nih.gov/protein/WP_009893147.1?report=genbank&log$=prottop&blast_rank=1&RID=C6UEZ061013) | 779/779 (96.48%) | 0.0 |
| phiCDKH02_69 | 55336-55151 | - | 61 | transcriptional regulator | [AbrB/MazE/SpoVT family DNA-binding domain-containing protein [*Clostridioides difficile*]](https://blast.ncbi.nlm.nih.gov/Blast.cgi#alnHdr_HBF8536590) | [HBF8536590.1](https://www.ncbi.nlm.nih.gov/protein/HBF8536590.1?report=genbank&log$=prottop&blast_rank=2&RID=C6V1872G016) | 60/61 (98.36%) | 2e-34 |
| phiCDKH02_70 | 55693-55565 | - | 42 | hypothetical protein | hypothetical protein [Clostridioides difficile] | [WP_003433683.1](https://www.ncbi.nlm.nih.gov/protein/WP_003433683.1?report=genbank&log$=prottop&blast_rank=1&RID=C6VAMPDK013) | 42/42 (100%) | 4e-18 |

^a^Only the most relevant hit is shown
