# Supplementary material for: The phi027 bacteriophage influences physiology and virulence of the lysogenic strain of Clostridioides difficile
Source: Sci Rep. 2025 May 29;15:18856. doi: 10.1038/s41598-025-04106-0 (PMC12122855; doi:10.1038/s41598-025-04106-0)
Supplement: Supplementary file 5 — Supplementary Material 5 [file 41598_2025_4106_MOESM5_ESM.docx]

**Table S2.** Bacterial strains and plasmids used in this study.

| **Strains and Plasmids** | **Relevant characteristics** | | | **Reference or source** | **Accession no** |
| --- | --- | --- | --- | --- | --- |
| **Strains** |  | | |  |  |
| ***E. coli*** |  | | |  |  |
| DH5α | *F- gyrA96 recA1 relA1 endA1 thi-1 hsdR17 glnV44 deoR D (lacZYA-argF) U169[f80dD(lacZ)M15]* | | | [1] |  |
| **sExpress** | NEB Express (R702): *fhuA2 [lon] ompT gal sulA11 R(mcr-73::miniTn10--TetS)2 [dcm] R(zgb-210::Tn10--TetS) endA1 Δ(mcrC-mrr)114::IS10* | | | [2] |  |
| ***C. difficile*** |  | **Ribotype** | **Isolation date** |  |  |
| 500/12 | Clinical strain | 176 | 2012 | MUW^a^ [3] | CP180209 |
| CKH08 | Derived from 500/12, Δ*phi027* |  |  | This work | CP180211 |
| 137/12 | Clinical strain | 027 | 2012 | MUW [3] | JBCJLK000000000 |
| 1974/12 | Clinical strain | 176 | 2012 | MUW [3] | JBCJLI000000000 |
| 2163/12 | Clinical strain | 027 | 2012 | MUW [3] | JBCJLH000000000 |
| 2282/12 | Clinical strain | 027 | 2012 | MUW [3] | JBCJLG000000000 |
| 3136/12 | Clinical strain | 176 | 2012 | MUW [3] | JBCJLF000000000 |
| 3290/12 | Clinical strain | 176 | 2012 | MUW [3] | JBCJLM000000000 |
| 700/13 | Clinical strain | 027 | 2013 | MUW [3] | JBCJLJ000000000 |
| OR/03/B | Clinical strain | 027 | 2023 | PROPHDIFF^b^ MUW | JBCJLL000000000 |
| KB/04/B | Clinical strain | 027 | 2023 | PROPHDIFF MUW | JBCJLE000000000 |
| WL/14/B | Clinical strain | 027 | 2023 | PROPHDIFF MUW | JBCJLR000000000 |
| SL/15/Ba | Clinical strain | 027 | 2023 | PROPHDIFF MUW | JBCJLN000000000 |
| BM/25/D | Clinical strain | 027 | 2022 | PROPHDIFF MUG^c^ | JBCJLO000000000 |
| AA/01/By | Clinical strain | 027 | 2022 | PROPHDIFF MUS^d^ | JBCJLQ000000000 |
| SM/02/By | Clinical strain | 027 | 2022 | PROPHDIFF MUS | JBCJLP000000000 |
|  |  | | |  |  |
| **Plasmids** |  | | |  |  |
| pUC19 | *ori(pMB1) lacZ* Amp^R^ | | | [4] |  |
| pEcCdH01 | Derived from pUC19, *sRNAP::crRNA* (23-nt repeat), PCR template for generating *sRNAP::crRNA* for retargeting | | | This work |  |
| pWH34 | Derived from pMTL82151, *E. coli-C. difficile* shuttle vector, *iLacP::AsCpf1,* BtgZI-BtgZI double sites, “Chassis” plasmid for gene-targeting plasmid construction, Cm^R^/Tm^R^ | | | [5] |  |
| pEcCdH07 | Derived from pWH34, targeting *phi027* with one spacer, Cm^R^/Tm^R^ | | | This work |  |
| pEcCdH08 | Derived from pWH34, targeting *phi027* with two sets of spacers, Cm^R^/Tm^R^ | | | This work |  |

^a^ Collection of *C. difficile* strains of the Department of Medical Microbiology, Medical University of Warsaw, Poland

^b^ PROPHDIFF: *C. difficile* strains were obtained as part of the implementation of the Polish National Science Centre project No. 2021/43/B/NZ6/00461

^c^ Collection of *C. difficile* strains of the Division of Molecular Bacteriology, Medical University of Gdańsk, Poland

^d^ Collection of *C. difficile* strains of the Department of Medical Microbiology, Faculty of Medical Science in Katowice, Medical University of Silesia, Poland

**References**

[1] Woodcock DM, Crowther PJ, Doherty J, Jefferson S, DeCruz E, Noyer-Weidner M, Smith SS, Michael MZ, Graham MW. Quantitative evaluation of *Escherichia coli* host strains for tolerance to cytosine methylation in plasmid and phage recombinants. Nucleic Acids Res. 1989, 17:3469-78.

[2] Woods C, Humphreys CM, Rodrigues RM, Ingle P, Rowe P, Henstra AM, Köpke M, Simpson SD, Winzer K,  Minton NP. A novel conjugal donor strain for improved DNA transfer into *Clostridium* spp. Anaerobe 2019, 59:184-191.

[3] Pituch H, Obuch-Woszczatyński P, Lachowicz D, Wultańska D, Karpiński P, Młynarczyk G, van Dorp SM, Kuijper EJ Hospital-based *Clostridium difficile* infection surveillance reveals high proportions of PCR ribotypes 027 and 176 in different areas of Poland, 2011 to 2013. (Polish *Clostridium difficile* Study Group.). Euro Surveill. 2015; 20(38).

[4] Yanisch-Perron C, Vieira J, Messing J. Improved M13 phage cloning vectors and host strains: nucleotide sequences of the M13mp18 and pUC19 vectors. Gene 1985, 33:103-19.

[5] Hong W, Zhang J, Cui G, Wang L, Wang Y. Multiplexed CRISPR-Cpf1-Mediated Genome Editing in *Clostridium difficile* toward the Understanding of Pathogenesis of C. difficile Infection. ACS Synth Biol 2018, 7:1588-1600.
