# Supplementary material for: The phi027 bacteriophage influences physiology and virulence of the lysogenic strain of Clostridioides difficile
Source: Sci Rep. 2025 May 29;15:18856. doi: 10.1038/s41598-025-04106-0 (PMC12122855; doi:10.1038/s41598-025-04106-0)
Supplement: Supplementary file 7 — Supplementary Material 7 [file 41598_2025_4106_MOESM7_ESM.docx]

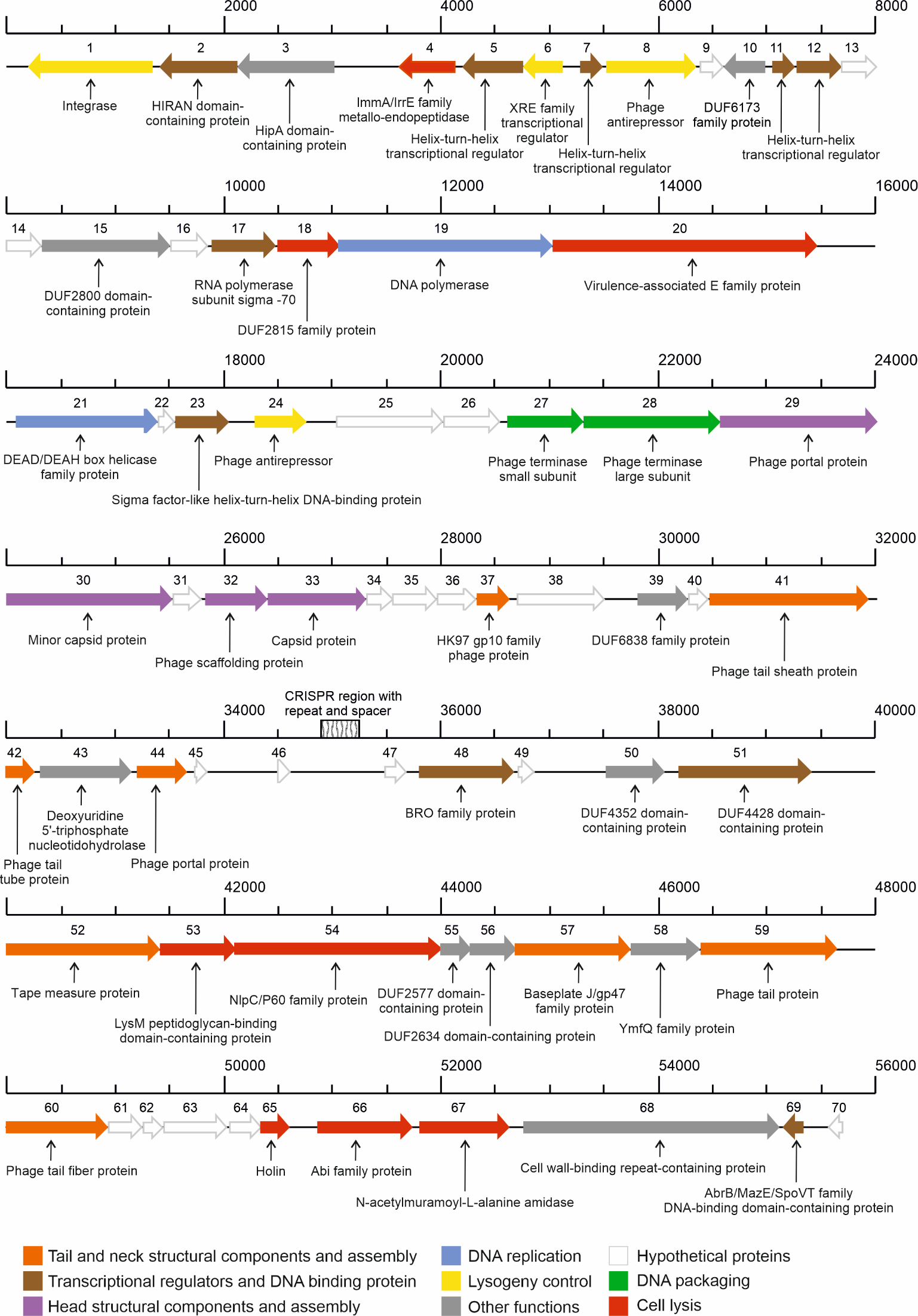


**Figure S2**. Features of the phage phiCDKH02 genome. The predicted ORFs and their orientations are represented by arrows. The putative functional assignments are indicated below the ORFs. The functional modules were assigned based on gene annotation and genomic organization and are shown in different colours. The position of the CRISPR region with its repeats and spacers is indicated by diagonal hatching.
