# Supplementary figures and images for: The phi027 bacteriophage influences physiology and virulence of the lysogenic strain of Clostridioides difficile
Source: Sci Rep. 2025 May 29;15:18856. doi: 10.1038/s41598-025-04106-0 (PMC12122855; doi:10.1038/s41598-025-04106-0)

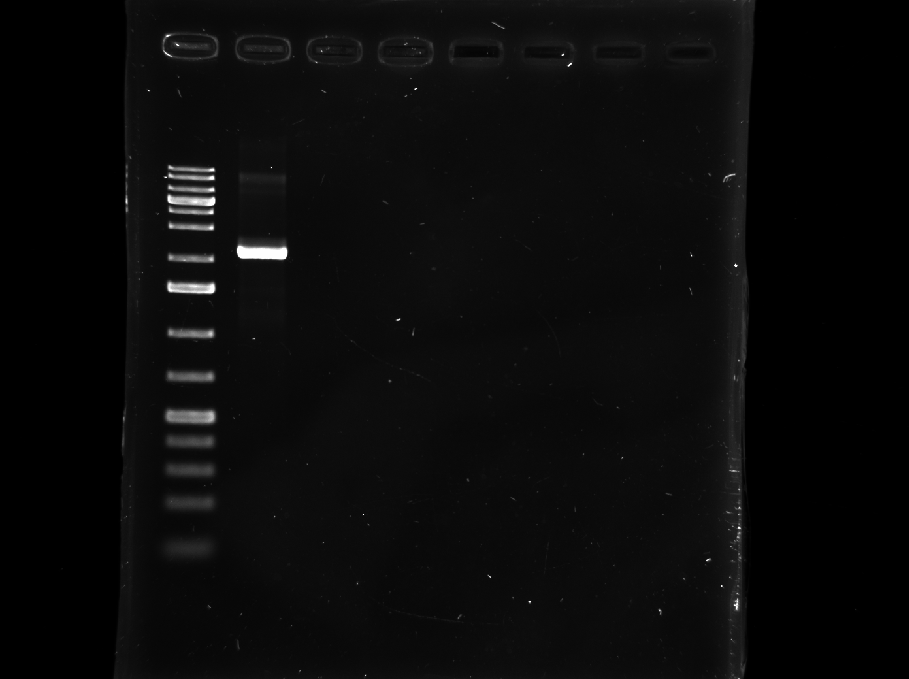

Supplement: Supplementary file 8 — Supplementary Material 8 [file 41598_2025_4106_MOESM8_ESM.tif]

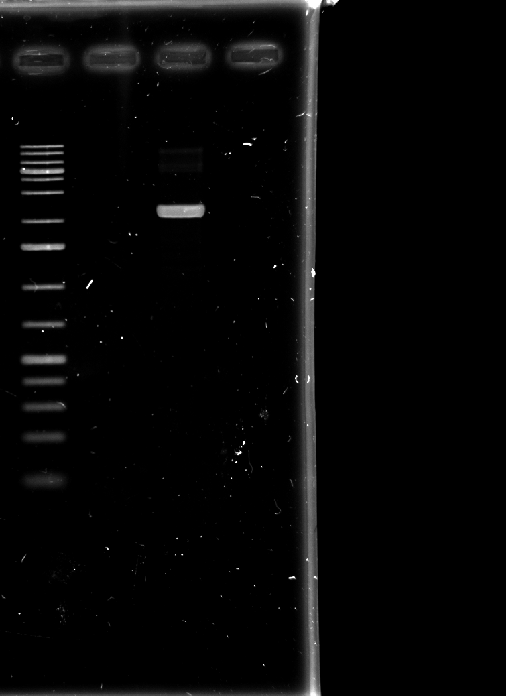

Supplement: Supplementary file 9 — Supplementary Material 9 [file 41598_2025_4106_MOESM9_ESM.tif]

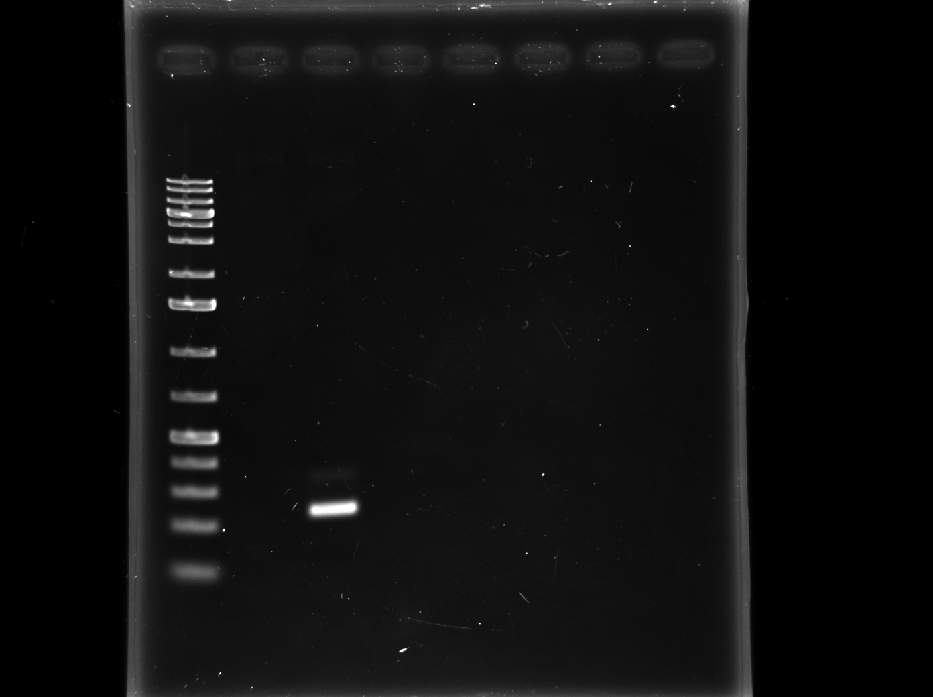

Supplement: Supplementary file 10 — Supplementary Material 10 [file 41598_2025_4106_MOESM10_ESM.tif]
