# Supplementary material for: The phi027 bacteriophage influences physiology and virulence of the lysogenic strain of Clostridioides difficile
Source: Sci Rep. 2025 May 29;15:18856. doi: 10.1038/s41598-025-04106-0 (PMC12122855; doi:10.1038/s41598-025-04106-0)
Supplement: Supplementary file 11 — Supplementary Material 11 [file 41598_2025_4106_MOESM11_ESM.docx]

**Table S3.** Primers used in this study.

| **Primers** | **Sequences (5’-3’)** |
| --- | --- |
| CDHmyoF | TATACCAGAGCAGTTRCTRA |
| CDHmyoR | CMTCCTTCAAYTGTTTGTAA |
| CDHsiphoF | TTATGCGCTTTGCTRTTYAA |
| CDHsiphoR | MGTTTTCATTGCTCCCATTT |
| phiCDKH02_1F | CATTCATATGATACTAGT |
| phiCDKH02_1R | GTAGATTTATGCTAATTTC |
| phiCDKH02_2F | TTCTCTTCTCGTAATTGTTTG |
| phiCDKH02_2R | ACTTCTTTTCTTCATGTTCTTC |
| phiCDKH02_3F | TTATTTGGAGGTATTTCATGG |
| phiCDKH02_3R | CTCТTАTTTTGТССТТТССАG |
| YW3105 | AAAGTTAAAAGAAGAAAATAGAAATATAATCTTTAATTTGAAAAGATTTA |
| phi500_S1_0 | ATTATAATACGGTGGCAAATGGGTGGCATCTACAAGAGTAGAAATTAATGGTGGAATGATAAGGGTT |
| phi500_S1S2_0 | ATTAGACTTAGCAAATGGTGTTGCTGAATCTACAAGAGTAGAAATTATAATACGGTGGCAAATGGGTGGC |
| phi500_S1_1 | AGATGCCACCCATTTGCCACCGTATTATAATTTCTACTCTTGTAGATTTTAGTGATAGAGTGATAAGTAAAA |
| phi500_S1S2_1 | GATTCAGCAACACCATTTGCTAAGTCTAATTTCTACTCTTGTAGATTTTAGTGATAGAGTGATAAGTAAAA |
| phi500_S1_2 | ATTATTATTTACTTAAGTTGTAATATATTTACTTAAGTTGTAATACTATT |
| phi500_S1_3 | AATAGTATTACAACTTAAGTAAATATATTACAACTTAAGTAAATAATAAT |
| phi500_S1_4 | CTCCATGGACGCGTGACGTCGACTCATTCCAACTATACCAGTTTATAGGA |
| ilvB-F | GGC TTG GAA ATA TAG ATA GAA AG |
| cotG-R | CCA ATG ACA TGT TGA ATA TCC |
| phi027-F | TTATTTGGAGGTATTTCATGG |
| phi027-R | CTCTTATTTTGTCCTTTCCAG |
| edit-F | GACCCATCTGAAATAAGTAAAAATATAG |
| int-R | TATAGCTAGCATGAAAGGTGGAGTAAGAAAAAG |
